# Expression Pattern of the AB1-Gal4 Driver in *Drosophila* Third-Instar Larvae

**DOI:** 10.3390/ijms26093923

**Published:** 2025-04-22

**Authors:** Anna A. Ogienko, Evgeniya N. Andreyeva, Lyubov A. Yarinich, Alexey V. Pindyurin, Nadezhda V. Battulina, Evgeniya S. Omelina

**Affiliations:** Institute of Molecular and Cellular Biology of the Siberian Branch of the Russian Academy of Sciences (IMCB SB RAS), 630090 Novosibirsk, Russia

**Keywords:** *Drosophila*, Gal4/UAS, P{GawB}, AB1-Gal4, driver, salivary glands, CNS, photoreceptors, motor neurons

## Abstract

*Drosophila* has provided a highly attractive model system for studying various tissue- and stage-specific processes as well as their pathologies, including a range of human diseases. The existence of a large number of diverse Gal4 drivers to precisely control the expression patterns of UAS transgenes simplifies such studies. However, the choice of driver is always critical, as its possible ectopic expression in non-target cells and tissues can directly impact the results. Therefore, it is very important to thoroughly characterize both the molecular nature and expression pattern of each Gal4 driver line. Here, we aim to fill such gaps regarding the AB1-Gal4 driver, which is typically used to express UAS transgenes in larval salivary glands. In this fly line, the P{GawB} enhancer trap construct encoding the Gal4 protein resides within overlapping evolutionary conserved *spastin* (*spas*) and *Mitochondrial Rho* (*Miro*) genes. Both these genes are expressed in a number of tissues, including the central nervous system (CNS), and their human orthologs are associated with neurodegenerative diseases. Consistently, we demonstrate that, in third-instar larvae, the expression pattern of AB1-Gal4 is also not restricted to salivary glands. We detect its activity in a subset of Elav-positive neurons in the CNS, including motor neurons, as well as in specific photoreceptor cells in eye discs.

## 1. Introduction

The Gal4/UAS system is one of the most powerful tools for targeted gene expression. It is based on the properties of the yeast Gal4 transcription factor, which activates transcription of the target genes by binding to UAS regulatory sequences in their promoters. The introduction of the binary Gal4/UAS system in *Drosophila* [1,2,3,4] enabled the expression of constructs for RNAi [5,6,7], Cre and FLP recombinases [8,9], components of the CRISPR/Cas systems [10,11,12,13], and various genes of interest (e.g., human disease genes, longevity genes, their mutant variants, etc.) in a controlled spatiotemporal manner [14,15]. The expression patterns of UAS transgenes restricted to a certain cell type or tissue and determined by the developmental stage of the fly are achieved by using appropriate Gal4 drivers. The latter can be subdivided into two groups. First, these are transgenes expressing the Gal4 transcriptional activator under the control of different specific promoters/enhancers, which are integrated at random or predefined genomic locations. Second, these are so-called enhancer trap transposons carrying a Gal4 coding sequence downstream of a minimal promoter or even without it at all, wherein expression patterns are primarily defined by their position in the genome [4,16]. Importantly, for some experimental setups, the choice of Gal4 driver is crucial, as it can significantly influence the results and/or their interpretation. Currently, many thousands of different drivers are available from *Drosophila* stock centers and private collections. Particularly, a large variety of fly lines were generated for Gal4 expression in neuronal and glial cells of the central nervous system (CNS), different somatic cells, germline stem cells, and imaginal discs (http://flybase.org/GAL4/freq_used_drivers/, accessed on 17 April 2025). Among those is also a large set of drivers derived from the P{GawB} enhancer trap element consisting of (i) a minimal functional 5′ end of the *P*-element (including the *P*-transposase promoter), (ii) a fragment of the *Drosophila Hsp70* 5′ UTR, (iii) the yeast Gal4 coding sequence, (iv) a fragment of the yeast Gal4 transcriptional terminator followed by a fragment of the *Drosophila Hsp70* 3′ UTR, (v) a mini-*white* reporter gene driven by the *Drosophila Hsp70* promoter, (vi) a plasmid vector backbone, and (vii) the 3′ end of the *P*-element [4,16]. It was shown that many P{GawB}-carrying drivers are expressed in larval salivary glands, which is possibly due to the presence of a cryptic salivary gland enhancer within the *Hsp70* 5′ UTR sequence [17,18,19,20].

One of the P{GawB}-derived drivers is AB1-Gal4 [21], which, according to FlyBase, has been used in more than 50 studies (http://flybase.org/reports/FBti0001249.html, accessed on 17 April 2025) [22,23,24]. Among these studies, we found two indirect indications that the expression pattern of the AB1-Gal4 driver might be not limited to larval salivary glands. First, it was demonstrated that UAS-*hid* expression activated by AB1-Gal4 results in larval death [25]. In contrast, complete ablation of the salivary glands caused by UAS-*hid* expression driven by another P{GawB}-derived driver, *Hsp70*-Gal4, allowed larvae to survive until puparium formation. Thus, AB1-Gal4 likely provides expression of the apoptosis-inducer gene *hid* not only in salivary glands but also in some vital tissues [25]. Second, AB1-Gal4 was identified as “an adult CNS driver”, and it was shown that this driver can rescue the maturation of rhodopsin, restoring its chromophore formation [26], through UAS-*ninaB* activation in *ninaB* null mutants like neuronal-specific *elav*-Gal4 (C155) and V55-Gal4 drivers do [27]. Interestingly, the *ninaB* gene is expressed exclusively in the adult brain [28], which indirectly suggests that AB1-Gal4 might be active in this tissue. However, the abovementioned works did not provide any direct (e.g., cytological) evidence of the AB1-Gal4 driver expression in tissues other than larval salivary glands. Thus, the complete expression pattern of AB1-Gal4 is not currently known, nor is the molecular nature of this driver (both the complete sequence of the P{GawB} transposon and its genomic insertion site).

Previously, we have shown that *Drosophila* driver lines should be thoroughly characterized before use, as they could be more complex in genetic composition than expected, which can affect the experimental outcome. For instance, fly lines can carry uncharacterized transposon constructs or their remnants, sometimes residing nearby or within genes involved in the process or pathway under study, that can directly or indirectly affect the expression pattern of the UAS reporter gene [29,30].

In the present study, we identified the precise genomic location of the P{GawB} transposon and clarified its complete sequence in the BDSC fly line #1824 carrying AB1-Gal4. Although the P{GawB} insertion resides simultaneously within two overlapping genes, *Mitochondrial Rho* (*Miro*) and *spastin* (*spas*), their expression levels in third-instar larval salivary glands and CNS seem to be not affected. Also, we found that in third-instar larvae, in addition to the salivary glands, the AB1-Gal4 driver is active in the CNS, namely, in the central brain, the ventral nerve cord (VNC), the optic lobes, and in certain photoreceptor cells of the eye discs. We carefully characterized the driver expression pattern in the CNS and eye discs using UAS-GFP reporters and demonstrated that it partially colocalizes with Elav-positive neurons. In particular, AB1-Gal4 is expressed in the neurons of the VNC, including motor neurons, whereas in the eye discs, its expression begins only in the R8 photoreceptors and later expands to some other photoreceptors. The described features of the AB1-Gal4 driver should be taken into account both for designing experimental setups with its usage and in interpretation of their results.

## 2. Results

### 2.1. Molecular Analysis of the AB1-Gal4 Driver Line

**Figure 1:**
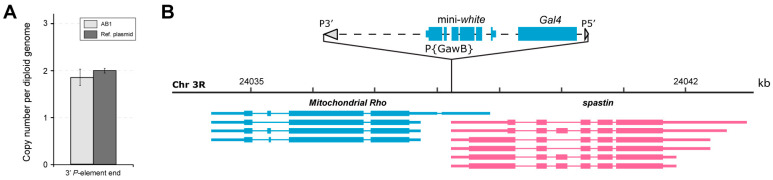
The absence of extra *P*-element-based transposons and genomic location of P{GawB} in the AB1-Gal4 driver line. (**A**) Only one *P*-element transgene per haploid genome was detected in AB1-Gal4 homozygous flies by qPCR (no statistically significant difference was found between the reference plasmid pP5′-Vps36-759bp-P3′ [29], carrying single copies of the 3′ *P*-element end and a fragment of the *Vps36* gene used for normalization, and the fly genomic DNA according to pairwise *t*-test at significance level *p* < 0.05). The experiment was conducted in two replicates. Error bars represent standard errors of the mean. AB1 denotes AB1-Gal4 homozygotes. (**B**) Genomic region around the transposon insertion site. Genes on the forward and reverse strands are shown in magenta and blue, respectively. Coding sequences, UTRs, and introns are represented as wide bars, narrow bars, and lines, respectively. Gray triangles represent the 5′ and 3′ *P*-element ends. The P{GawB} transposon is not shown to scale.

Since we have previously found extra unexpected transgenes in a number of *Drosophila* driver lines [29,30], we first estimated the number of *P*-element-based transposons in the genome of the BDSC fly line #1824 carrying AB1-Gal4. Only a single transposon was detected in this line according to the qPCR measurements (Figure 1A). Next, using an inverse-PCR approach [31], we identified that the *P*-element-based transposon is integrated at chr3R: 24,038,230–24,038,237 (BDGP Release 6 of the *D. melanogaster* genome assembly [32]). The genomic location was verified by PCR with primers specific to the mini-*white* gene present within the transposon and the genomic regions flanking the insertion site (Supplementary Figure S1). The identity and integrity of the P{GawB} transposon were confirmed by Sanger sequencing of the obtained PCR products. This also allowed us to determine the entire sequence of P{GawB} (GenBank #PV287694), and to fill a gap and correct some potential inaccuracies in the previously compiled sequence of this construct (http://flybase.org/api/sequence/id/FBtp0000352/compiled, accessed on 17 April 2025; Supplementary File S1). The P{GawB} transposon resides simultaneously within two overlapping genes, *Mitochondrial Rho* (*Miro*) [33,34] and *spastin* (*spas*) [35,36]. More specifically, the 5′ UTR of all *spas* transcripts and the 5′ UTR of the longest *Miro* transcript are affected (Figure 1B).

### 2.2. Analysis of the Influence of the P{GawB} Transposon Insertion on the Expression of the Miro and Spas Genes in the Third-Instar Larval Salivary Glands and CNS

*Miro* is known to encode mitochondrial rho GTPase playing multiple roles in regulating the transport of mitochondria, particularly in the nervous system and spermatogenesis [37,38,39]. Spas is a member of the AAA ATPase family associated with diverse cellular activities and contains a microtubule-interacting domain, suggesting an active role in cytoskeleton interactions [40]. It plays a role in the organization of the microtubule cytoskeleton in neurons, and the loss of this Spas function leads to defects in synaptic growth and neurotransmission [40,41].

**Figure 2:**
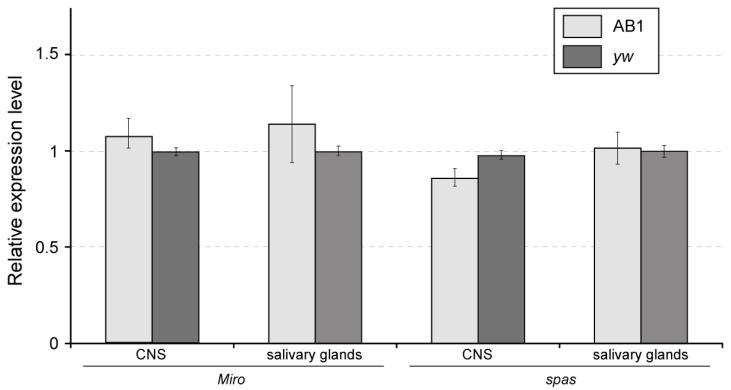
Expression levels of the *Miro* and *spas* genes (measured by RT-qPCR) in the third-instar larval salivary glands and CNS. Relative mRNA levels were normalized to those of the reference genes *RpL32* and *robl*. No statistically significant differences were observed between the AB1-Gal4 and control *yw* animals (pairwise *t*-test at significance level *p* < 0.05). The experiment was conducted in two replicates. Error bars represent the standard errors of the mean. AB1 denotes AB1-Gal4 homozygotes.

The expression patterns of the *Miro* and *spas* genes in third-instar larvae are quite diverse. *Miro* is expressed in the CNS, salivary glands, digestive system, Malpighian tubules, fat body, trachea, and imaginal discs [42,43,44]. The *spas* mRNA was detected in imaginal discs, the CNS, salivary gland, digestive system, fat body, and carcass [43]. So, none of these genes is expressed exclusively in salivary glands. In fact, the expression level of each gene is higher in imaginal discs and the CNS compared to salivary glands [43]. Importantly, we found no statistically significant difference in the transcription levels of the *Miro* and *spas* genes in the third-instar larval salivary glands and CNS between the AB1-Gal4 driver line and a wild-type control (Figure 2). These results are consistent with the Western blot analysis performed on the third-instar larval CNS (Supplementary Figure S2). Thus, the P{GawB} insertion within the shared regulatory region of the *Miro* and *spas* genes does not affect their bulk expression levels in the third-instar larval salivary glands and CNS.

### 2.3. Expression Pattern of the AB1-Gal4 Driver in the CNS of Third-Instar Larvae

**Figure 3:**
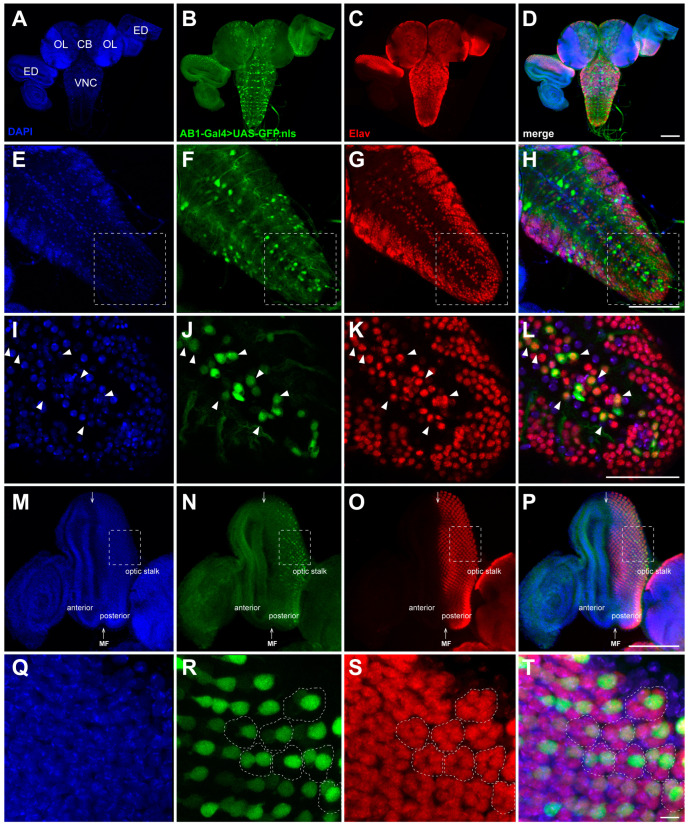
Comparison of the nuclear GFP expression pattern elicited by the AB1-Gal4 driver with the Elav immunostaining patterns in the CNS and eye discs from third-instar larvae. Maximum intensity projections of confocal images of tissues stained with anti-Elav antibodies are shown. (**A**–**D**) Overlap of the GFP expression pattern and Elav localization in the CNS and eye discs. (**E**–**H**) Partial colocalization of the GFP and Elav expression patterns in the VNC. GFP is expressed only in a subset of Elav-positive cells in the VNC. (**I**–**L**) Part of the VNC enlarged from (**E**–**H**), highlighted with a dotted rectangle. Arrowheads indicate examples of GFP-positive neurons that colocalize with Elav. (**M**–**P**) Partial colocalization of the GFP and Elav expression patterns in the posterior part of the eye disc. (**Q**–**T**) Part of the eye disc enlarged from (**M**–**P**), highlighted with a dotted rectangle. The clusters of eight photoreceptor cells (ommatidia), visualized by Elav (curved dotted figure), contain more than one GFP-positive cell. CB—central brain; OL—optic lobe; ED—eye disc; VNC—ventral nerve cord; MF—morphogenetic furrow. Scale bars: (**A**–**D**)—100 μm; (**E**–**H**)—100 μm; (**I**–**L**)—50 μm; (**M**–**P**)—100 μm; (**Q**–**T**)—5 μm.

We found that the AB1-Gal4 driver is expressed not only in the salivary glands but also in the larval CNS and eye discs, as well as in the trachea and male gonads. We carefully examined the expression pattern of the AB1-Gal4 driver in the CNS and eye discs using a nuclear GFP reporter (UAS-GFP.nls) and staining with specific antibodies (Figure 3). We detected a clear GFP signal localized in the cell nuclei of the central brain, the VNC, the optic lobes, and some cells of the eye discs (Figure 3). Furthermore, the AB1-Gal4 expression pattern partially overlapped with that of the neuronal marker Elav [45], indicating that this driver is specifically expressed in neurons (Figure 3). Previously, we have shown that another P{GawB}-containing driver 69B-Gal4 from the BDSC fly line #1774 is active in the CNS and has a similar intersection with the Elav expression pattern [30].

**Figure 4:**
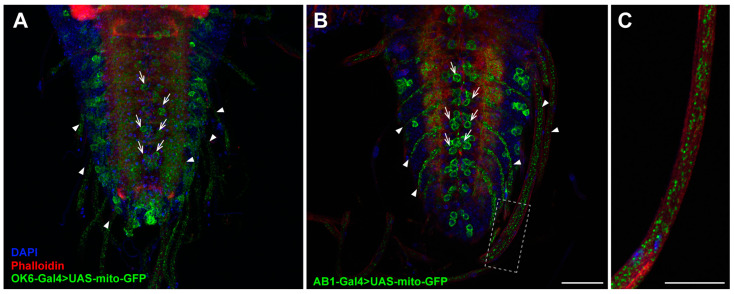
Comparison of the mito-GFP expression pattern elicited by the OK6-Gal4 and AB1-Gal4 drivers in the VNC of third-instar larvae. Maximum intensity projections of confocal images of tissues stained with phalloidin (red) and DAPI (blue). (**A**) Larvae of the UAS-mito-GFP/OK6-Gal4; +/+ genotype. The distribution of GFP-tagged mitochondria is clearly observed in the bodies of motor neurons (arrows) and axons (arrowheads). (**B**) Larvae of the UAS-mito-GFP/+; AB1-Gal4/AB1-Gal4 genotype, stained as in (**A**). The distribution pattern of GFP-tagged mitochondria is similar to that in (**A**) but even more distinct and bright. (**C**) Enlarged section from (**B**), highlighted with a dotted rectangle. GFP-tagged mitochondria are clearly visible in the axons. Scale bars: (**A**,**B**)—50 μm; (**C**)—25 μm.

The *Drosophila* larval CNS consists of the brain (comprising two lobes) populated by higher-order neurons and the VNC that includes interneurons, sensory neurons, and motor neurons [46]. To determine the types of neurons in which the AB1-Gal4 driver is active in the VNC (Figure 3I–L), we compared its expression pattern with that of the OK6-Gal4 driver, which is known to express Gal4 specifically in motor neurons [47,48]. We used a GFP reporter fused with a mitochondrial targeting signal (UAS-mito-GFP), enabling the visualization of not only motor neuron cell bodies but also the axons extending from these motor neurons. We found that the expression pattern of the UAS-mito-GFP reporter under the control AB1-Gal4 is very similar to that elicited by the OK6-Gal4 driver (Figure 4). This suggests that one of the subpopulations of neurons in which AB1-Gal4 is expressed consists of motor neurons. Additionally, the mitochondrial transport seems to be not impaired in AB1-Gal4 homozygous larvae, as we did not observe any difference in mitochondrial localization in the axons compared to the control (Figure 4).

### 2.4. Expression Pattern of the AB1-Gal4 Driver in Eye Discs of Third-Instar Larvae

The *Drosophila* compound eye consists of ommatidia, which begin to form as regularly spaced clusters of photoreceptors (R1–R8) in the eye discs of third-instar larvae [49]. R8 is the first to differentiate, and then, through cell–cell interactions, it induces the differentiation of other photoreceptors and some accessory cells that join the cluster in a specific sequence [50,51,52,53,54]. The differentiation of the regularly spaced R8 begins in a single row at the posterior edge of the eye disc, which is influenced by signals from the morphogenetic furrow (MF) that crosses the disc along the dorso-ventral axis. Subsequently, the clusters of the R8, R2, and R5 photoreceptors induce the progression of the MF and the wave of differentiation towards the anterior part of the disc through a positive feedback loop [49,55]. By the late third-instar larval stage, the MF has crossed about half of the eye disc (Figure 3M), which contains photoreceptor clusters at different stages of formation, with more mature clusters located at the posterior edge [50]. The R1–R6 cells represent the major class of photoreceptors in the retina and project their axons to the first optic lobe, known as the lamina. The R7/R8 photoreceptors project their axons to the second optic lobe, the medulla [56,57].

**Figure 5:**
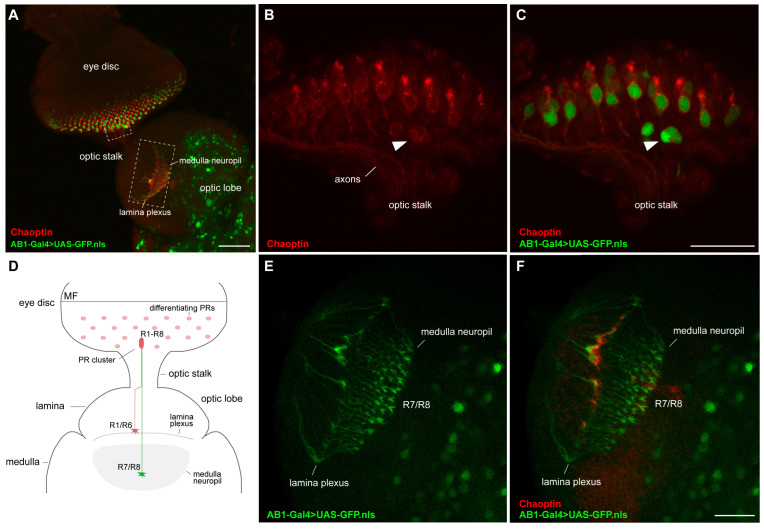
Expression of AB1-Gal4 in the optic lobe and eye discs from third-instar larvae. (**A**,**B**,**E**) Maximum intensity projections of confocal images of AB1-Gal4>UAS-GFP.nls third-instar larval optic lobe and eye discs stained with anti-Chaoptin antibodies and showing GFP expression driven by the AB1-Gal4 driver. (**B**,**C**) A section of the eye disc is enlarged from (**A**), highlighted with a small dotted rectangle. An arrowhead indicates a cluster with more than one photoreceptor exhibiting AB1-Gal4 expression. (**D**) Schematic morphology of the third-instar larval optic lobe and eye disc in frontal orientation. PR—photoreceptor. (**E**,**F**) A section of the optic lobe is enlarged from (**A**), highlighted with a large dotted rectangle. R7/R8 axons terminate in the medulla, while R1–R6 axons form the lamina plexus. Scale bars: (**A**)—50 μm; (**B**,**C**)—20 μm; (**E**,**F**)—20 μm.

We have found that the AB1-Gal4 expression pattern partially overlaps with that of the neuronal marker Elav in the posterior part of the eye discs (Figure 3M–P) and is observed in more than one cell per cluster of photoreceptors (Figure 3Q–T). To determine in which type of photoreceptors AB1-Gal4 is expressed, we immunolabeled the photoreceptors of the AB1-Gal4>UAS-GFP.nls third-instar larvae with antibodies against the Chaoptin protein. Chaoptin is a photoreceptor cell-specific glycoprotein that is bound to the cell and axon membranes via a glycosylphosphatidylinositol anchor [58,59,60]. Staining with anti-Chaoptin antibodies revealed that in the AB1-Gal4>UAS-GFP.nls eye discs, most of the GFP-positive cells direct their axons to the medulla of the optic lobe, similar to R7/R8, while some GFP-positive cells likely project their axons to form the neural plexus at the lamina similar to R1–R6 (Figure 5).

**Figure 6:**
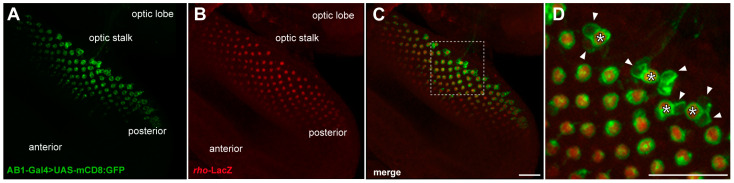
The AB1-Gal4 driver is initially expressed in R8 and later in some other photoreceptors. Maximum intensity projections of confocal images of the part of the third-instar larval eye disc, stained with anti-beta-galactosidase (LacZ) antibodies and showing GFP expression driven by the AB1-Gal4 driver. (**A**) The AB1-Gal4 expression pattern in photoreceptors, revealed by the UAS-mCD8:GFP reporter, is clearly detected in the posterior margin of the eye disc. (**B**) R8 photoreceptors are indicated by the expression of *rho*-LacZ, which is restricted mainly to the developing R8 [61]. (**C**) Colocalization of *rho*-LacZ and AB1-Gal4>UAS-mCD8:GFP. (**D**) Enlarged section from (**C**), highlighted with a dotted square. Asterisks indicate R8 photoreceptors. Arrowheads indicate GFP-positive photoreceptors that do not colocalize with the R8-specific marker LacZ. Scale bar: 20 μm for all images.

To identify in which photoreceptor the expression of AB1-Gal4 is first detected, we immunostained the eye discs of third-instar larvae of the UAS-mCD8:GFP/+; AB1-Gal4/*rho*-lacZ genotype, expressing the membrane-targeted GFP, with antibodies against beta-galactosidase (LacZ). We observed complete colocalization of the R8-specific marker *rho*-LacZ [61] with GFP-positive photoreceptors (Figure 6), but the GFP pattern was wider (Figure 6D), which may indicate that AB1-Gal4 is active not only in R8 photoreceptors, but also probably in some other photoreceptors.

## 3. Discussion

The ability to manipulate small subsets of neurons is critical to many of the experimental approaches used to study neuronal circuits in genetic model organisms. In *Drosophila*, numerous genetic lines expressing the exogenous transcription factor Gal4 in different subsets of neurons are available [62,63]. The Gal4 protein drives the expression of reporter genes placed downstream of UAS, which are typically provided as separate transgenic constructs [1,4]. This modular approach has proven to be very powerful but depends on fly lines with clearly described Gal4 expression patterns, which are limited for specific subsets of cells.

In this study, we have characterized the expression pattern of the widely used salivary-gland-specific AB1-Gal4 driver [21], in the third-instar larval CNS, using GFP reporters with different intracellular localization. This expression pattern was compared to that of the nuclear Elav protein, which is exclusively expressed in neurons [64], and to the photoreceptor neuron-specific plasma membrane-associated marker Chaoptin [65]. We found that the AB1-Gal4 driver, in addition to being expressed in the salivary glands, is also active in the neurons of the central brain and VNC, as well as in part of the photoreceptors of the eye discs of third-instar larvae. Alongside other neurons in the VNC, AB1-Gal4 is expressed in the cell bodies and axons of motor neurons. In the eye discs, AB1-Gal4 is initially expressed in R8 photoreceptors and is later detected in some other photoreceptors.

The P{GawB} transposon, like all enhancer trap constructs, is expected to drive Gal4 expression in a genomic integration site-dependent manner [4,16,17]. In the AB1-Gal4 driver line, P{GawB} is inserted simultaneously within the two head-to-head overlapping genes *spas* and *Miro*; thus, it may reflect some aspects of the expression of both these evolutionary conserved genes. Notably, during the third-instar larval stage, the expression pattern of *Miro* is much broader than that of AB1-Gal4; in addition to the common salivary glands, trachea, and CNS, it also includes the digestive system, Malpighian tubules, fat body, and imaginal discs [42,43,44]. Like AB1-Gal4, *Miro* is expressed in motor neurons in the larval CNS, but there are no data on its activity in other CNS cells. The expression pattern of *spas* is also broader than that of AB1-Gal4; in addition to the salivary glands and CNS, *spas* gene is also active in the digestive system, fat body, carcass, and motor neurons [40]. Thus, the regulation of AB1-Gal4 expression is not obvious. Most likely, besides the activity due to a cryptic salivary gland enhancer in P{GawB}, the expression of Gal4 might be also in part regulated by some enhancers located within *Miro* and *spas* or even more distant genes.

The wide range of Gal4 drivers active in specific cell types and subtypes of the *Drosophila* CNS, along with the conservation of genes across species and the potential for conducting rapid genetic analyses using the compact fly CNS, enables the use of *Drosophila* as a model for studying human neurodegenerative diseases. Since in the AB1-Gal4 line the insertion of the P{GawB} element does not seem to affect the expression or the functioning of the evolutionarily conserved *Miro* and *spas* genes in the larval CNS, this driver may be utilized for modelling certain human diseases in *Drosophila*. Specifically, it may be used to study mitochondrial transport, as disruptions in this process can lead to various human neurological and neurodegenerative disorders [66,67]. For instance, defects in mitochondrial movement are known to be associated with Parkinson’s disease (PD) and Alzheimer’s disease (AD) [68]. Interestingly, Miro is involved in interactions with PD- and AD-associated proteins [69,70,71], as well as in mitochondrial dynamics in neuronal axons crucial for the formation of long-term memory [72]. Defective mitochondrial axonal transport and Miro deficiency are also associated with another neurodegenerative disease: amyotrophic lateral sclerosis [73,74]. Additionally, AB1-Gal4 might be employed for the targeted reduction of *spas* expression in motor neurons and axons to model the genetic neurodegenerative syndrome spastic paraplegia 4, which is characterized by progressive muscle stiffness (spasticity) in the legs and difficulty walking [41,75,76].

Finally, we would like to note that it is a good practice to validate results using multiple driver lines that target the same cell type(s). The presence of a new variant of neuronal driver, carefully described at the molecular and cytological levels, might aid in studies of neurological diseases. We believe that the AB1-Gal4 driver may serve as an excellent tool for conducting research on neurodegenerative human diseases using the *Drosophila* model and for the development of new therapies for diseases related to mitochondrial dysfunction.

## 4. Materials and Methods

### 4.1. Fly Stocks

Flies were raised and crossed on standard cornmeal agar media at 25 °C. The fly stocks used in this study were obtained from the BDSC (Bloomington, IN, USA; https://bdsc.indiana.edu/, accessed on 17 April 2025): #1824 (y^1^ w^*^; P{*w^+mW.hs^* = GawB}AB1), referred to here as AB1-Gal4; #4775 (*w^1118^*; P{*w^+mC^* = UAS-GFP.nls}14), referred to here as UAS-GFP.nls; #52003 (*Miro^B682^*/TM6C, *Tb^1^ Sb^1^*); #95252 (w^*^; *Miro^Sd32^*/TM3, *Sb^1^ Ser^1^*); #64199 (P{*w^+mW.hs^* = GawB}OK6), referred to here as OK6-Gal4; #8442 (*w^1118^*; P{*w^+mC^* = UAS-mito-HA-GFP.AP}2/*CyO*), referred to here as UAS-mito-GFP; #32186 (w^*^; P{10XUAS-IVS-mCD8::GFP}attP40), referred to here as UAS-mCD8:GFP; #9231 (P{*ry^+t7.2^* = A92}*rho^BB02^ ry^506^*), referred to here as *rho*-lacZ; #6599 (*y^1^ w^67c23^*), referred to here as *yw*.

### 4.2. Determination of P-Element Transgene Copy Number and Mapping Its Insertion Site

Genomic DNA isolation, detection of *P*-element-based transgene copy number by qPCR, and mapping of the P{GawB} transposon insertion site using inverse-PCR [31] were performed as described previously [30].

### 4.3. Molecular Analysis of the P{GawB} Transposon

PCR products were obtained using Hot-Start Taq DNA polymerase (Biolabmix, Novosibirsk, Russia) with the following program: 95 °C for 30 s, followed by 35 cycles of 95 °C for 30 s, 58 or 60 °C for 60 s, and 72 °C for 5–7 min, with a final cycle at 72 °C for 5 min. For analysis of the 5′ half of P{GawB}, the primers AB1-ins-R1 (5′-ACTGGTTTTTAGTGCGTACCATCG-3′) and mWhite-qPCR-F1 (5′-AGGGGATCTCAAATATCAACTACAA-3′) were used (Supplementary Figure S1). For analysis of the 3′ half of P{GawB}, the primers AB1-ins-F1 (5′-AACATCGGATCCGAAACTGTCTGG-3′) and pUAST3 (5′-TATTCTGGTAGCTGTGCTCG-3′) were used (Supplementary Figure S1). The PCR products were column-purified and subjected to Sanger sequencing using the following primers: AB1-ins-F1, AB1-ins-R1, Amp-qPCR-R1 (5′-CACAGAAAAGCATCTTACGGATGG-3′), Ampicillin-R (5′-GATAAATCTGGAGCCGGTGAG-3′), GAL4-F (5′-CAACCAATTGCCTCCTCTAACG-3′), GAL4-F2 (5′-GATCCATTCAGCTTTCTCAGAATAC-3′), GAL4-F3 (5′-ACCCATAAAGACTCTACTCTCAAAC-3′), GAL4-R2 (5′-CTTGCTCGTCAAATGAGATTTAGC-3′), GAL4-R3 (5′-TCCACTTCAGTTCGAATCTTGTAAAG-3′), mH2B-eGFP-F1 (5′-GATTTAGAGCTTGACGGGGAAA-3′), mWhite-1 (5′-CGAACTCACTAGGAAAAGAAGTCG-3′), mWhite-2 (5′-CCAAAAAGATGAGGCCAATCAAGATG-3′), mWhite-3 (5′-CGACAACCATTTGAGGTATACTGG-3′), mWhite-4 (5′-TTGAGATGCATCTACACAAGGAAC-3′), mWhite-5 (5′-GTGACCTGTTCGGAGTGATTAG-3′), mWhite-6 (5′-CGAATTAATAGCTCCTGATCCTC-3′), mWhite-7 (5′-TCGCTGTGACACATACTTTCTGG-3′), mWhite-8 (5′-CAAATGTCAGCACACGATCATCG-3′), mWhite-qPCR-F1, pBS-F1 (5′-CAGGGTTTTCCCAGTCACGAC-3′), pBS-R1 (5′-GGCTTTACACTTTATGCTTCC-3′), pBS302-3 (5′-TCAGGGTTATTGTCTCATGAGC-3′), Pry2 (5′-CTTGCCGACGGGACCACCTTATGTTATT-3′), pTbStop-5 (5′-CTATGGAAAAACGCCAGC-3′), pTbStop-6 (5′-TTATCCGGTAACTATCGTCTTG-3′), pTbStop-7 (5′-ATCTTACCGCTGTTGAGATC-3′), pTbStop-11 (5′-GTTAGGCCACCACTTCAAG-3′), pUAST3, and qP5-R1 (5′-AGTGCACGTTTGCTTGTTGAG-3′).

### 4.4. RNA Extraction and RT-qPCR Analysis

Total RNA was isolated from the CNS and salivary glands of 60–90 third-instar larvae using RNAzol RT (Molecular Research Center, Cincinnati, OH, USA), as described previously [77]. For qPCR, the following primer pairs were used: Miro-qPCR-F4 (5′-TGGAAGAACAGGAGCTCACATC-3′) and Miro-qPCR-R4 (5′-GGTGACCGCATCGTTGTATATG-3′) specific for the *Miro* gene; spas-RT-qPCR-F2 (5′-ATTACGCCCCGAAGAAAATTGT-3′) and spas-RT-qPCR-R2 (5′-CGCAAACTGACAATTGGATCCA-3′) specific for the *spas* gene; robl-RT-qPCR-F1 (5′-TAGTGTCTGCCGTGTTTCCAAC-3′) and robl-RT-qPCR-R1 (5′-GTGGATTTGACCGGAATACCTTC-3′) specific for the reference *robl* gene; RPL32-qPCR-F1 (5′-CTAAGCTGTCGCACAAATGG-3′) and RPL32-qPCR-R1 (5′-AGGAACTTCTTGAATCCGGTG-3′) specific for the reference *RpL32* gene.

### 4.5. Western Blotting

Immunoblotting was performed as previously described [78]. The following primary antibodies were used: mouse monoclonal anti-α-Tubulin (1:4000; Sigma, St. Louis, MO, USA #T6199) and guinea pig anti-Miro (1:2000; #GP5; [37]). The primary antibodies were detected using polyclonal HRP-conjugated goat anti-mouse IgG (1:5000; Invitrogen, Waltham, MA, USA #G-21040) and polyclonal HRP-conjugated goat anti-guinea pig IgG (1:5000; Sigma #A7289), following the manufacturer’s protocols with the Novex ECL Chemiluminescent Substrate Reagent Kit (Thermo Fisher Scientific, Waltham, MA, USA). Images were captured using an Amersham Imager 600 System (GE Healthcare, Chicago IL, USA).

### 4.6. Immunostaining and Microscopy

The CNS and attached eye discs were dissected from third-instar larvae and fixed as described previously [30]. The following primary antibodies were used: polyclonal chicken anti-GFP (1:100; Thermo Fisher Scientific #PA1-9533), monoclonal mouse anti-Chaoptin (1:10; DSHB, Iowa City, IA, USA #24B10), monoclonal rat anti-Elav (1:10; DSHB #7E8A10), and monoclonal mouse anti-beta-galactosidase (1:10; DSHB #40-1a). They were detected using goat anti-rat IgG antibodies conjugated to Alexa Fluor 568 (1:500; Invitrogen #A-11077) and goat anti-mouse IgG antibodies conjugated to Alexa Fluor 568 (1:500; Invitrogen #A-11031), as well as goat anti-chicken IgY Alexa Fluor 488 (1:500; Invitrogen #A-11039). TRITC-labeled phalloidin (1:100, Sigma #P1951) was used to visualize F-actin, as described previously [79]. Finally, tissues were stained with 0.4 μg/mL DAPI dissolved in 1×PBS to detect DNA. All samples were imaged under the same settings using a confocal microscope LSM 710 (Carl Zeiss, Jena, Germany) with 10×/0.45 plan-apo and 20×/0.8 plan-apo lenses. Optical sections were combined using the LSM Image Browser version 4.2 software (Carl Zeiss).

## Data Availability

The original contributions presented in this study are included in the article/Supplementary Materials. Further inquiries can be directed to the corresponding author.

## Supplementary Materials

The following supporting information can be downloaded at: https://www.mdpi.com/article/10.3390/ijms26093923/s1.
