# Supplementary material for: Expression Pattern of the AB1-Gal4 Driver in Drosophila Third-Instar Larvae
Source: Int J Mol Sci. 2025 Apr 22;26(9):3923. doi: 10.3390/ijms26093923 (PMC12071433; doi:10.3390/ijms26093923)
Supplement: Supplementary file 1 [file ijms-26-03923-s001.zip › ijms-3584700-Supplementary File S1.pdf]

P{GawB} inserted in the AB1 line [Query, GenBank #PV287694] versus FlyBase-compiled sequence [Sbjct, http://flybase.org/api/sequence/id/FBtp0000352/compiled]

```
Identities: 11047/11294 (98%)
Gaps: 27/11294 (0%)

Query  1    CATGATGAAATAACATAAGGTGGTCCCGTCGATAGCCGAAGCT----TACCGAAGTATAC  56
            |||||||||||||||||||||||||||||||||||||||||||    |||||||||||||
Sbjct  1    CATGATGAAATAACATAAGGTGGTCCCGTCGATAGCCGAAGCTAGCTTACCGAAGTATAC  60

Query  57   ACTTAAATTCAGTGCACGTTTGCTTGTTGAGAGGAAAGGTTGTGTGCGGACGAATTTTTT  116
            ||||||||||||||||||||||||||||||||||||||||||||||||||||||||||||
Sbjct  61   ACTTAAATTCAGTGCACGTTTGCTTGTTGAGAGGAAAGGTTGTGTGCGGACGAATTTTTT  120

Query  117  TTTGAAAACATTAACCCTTACGTGCGGCCGCT-AAGCAAATAAACAAGCGCAGCTGAACA  175
            |||||||||||||||||||||||||||||||  ||||   | ||||||||||||||||||
Sbjct  121  TTTGAAAACATTAACCCTTACGTGCGGCCGCNNAAGC---TTAACAAGCGCAGCTGAACA  177

Query  176  AGCTAAACAATCTGCAGCCCAAGCTTGAAGCAAGCCTCCTGAAAGATGAAGCTACTGTCT  235
            ||||||||||||||||||||||||||||||||||||||||||||||||||||||||||||
Sbjct  178  AGCTAAACAATCTGCAGCCCAAGCTTGAAGCAAGCCTCCTGAAAGATGAAGCTACTGTCT  237

Query  236  TCTATCGAACAAGCATGCGATATTTGCCGACTTAAAAAGCTCAAGTGCTCCAAAGAAAAA  295
            ||||||||||||||||||||||||||||||||||||||||||||||||||||||||||||
Sbjct  238  TCTATCGAACAAGCATGCGATATTTGCCGACTTAAAAAGCTCAAGTGCTCCAAAGAAAAA  297

Query  296  CCGAAGTGCGCCAAGTGTCTGAAGAACAACTGGGAGTGTCGCTACTCTCCCAAAACCAAA  355
            ||||||||||||||||||||||||||||||||||||||||||||||||||||||||||||
Sbjct  298  CCGAAGTGCGCCAAGTGTCTGAAGAACAACTGGGAGTGTCGCTACTCTCCCAAAACCAAA  357

Query  356  AGGTCTCCGCTGACTAGGGCACATCTGACAGAAGTGGAATCAAGGCTAGAAAGACTGGAA  415
            ||||||||||||||||||||||||||||||||||||||||||||||||||||||||||||
Sbjct  358  AGGTCTCCGCTGACTAGGGCACATCTGACAGAAGTGGAATCAAGGCTAGAAAGACTGGAA  417

Query  416  CAGCTATTTCTACTGATTTTTCCTCGAGAAGACCTTGACATGATTTTGAAAATGGATTCT  475
            ||||||||||||||||||||||||||||||||||||||||||||||||||||||||||||
Sbjct  418  CAGCTATTTCTACTGATTTTTCCTCGAGAAGACCTTGACATGATTTTGAAAATGGATTCT  477

Query  476  TTACAGGATATAAAAGCATTGTTAACAGGATTATTTGTACAAGATAATGTGAATAAAGAT  535
            ||||||||||||||||||||||||||||||||||||||||||||||||||||||||||||
Sbjct  478  TTACAGGATATAAAAGCATTGTTAACAGGATTATTTGTACAAGATAATGTGAATAAAGAT  537

Query  536  GCCGTCACAGATAGATTGGCTTCAGTGGAGACTGATATGCCTCTAACATTGAGACAGCAT  595
            ||||||||||||||||||||||||||||||||||||||||||||||||||||||||||||
Sbjct  538  GCCGTCACAGATAGATTGGCTTCAGTGGAGACTGATATGCCTCTAACATTGAGACAGCAT  597

Query  596  AGAATAAGTGCGACATCATCATCGGAAGAGAGTAGTAACAAAGGTCAAAGACAGTTGACT  655
            ||||||||||||||||||||||||||||||||||||||||||||||||||||||||||||
Sbjct  598  AGAATAAGTGCGACATCATCATCGGAAGAGAGTAGTAACAAAGGTCAAAGACAGTTGACT  657

Query  656  GTATCGATTGACTCGGCAGCTCATCATGATAACTCCACAATTCCGTTGGATTTTATGCCC  715
            ||||||||||||||||||||||||||||||||||||||||||||||||||||||||||||
Sbjct  658  GTATCGATTGACTCGGCAGCTCATCATGATAACTCCACAATTCCGTTGGATTTTATGCCC  717
```

```
Query  716  AGGGATGCTCTTCATGGATTTGATTGGTCTGAAGAGGATGACATGTCGGATGGCTTGCCC  775
            ||||||||||||||||||||||||||||||||||||||||||||||||||||||||||||
Sbjct  718  AGGGATGCTCTTCATGGATTTGATTGGTCTGAAGAGGATGACATGTCGGATGGCTTGCCC  777

Query  776  TTCCTGAAAACGGACCCCAACAATAATGGGTTCTTTGGCGACGGTTCTCTCTTATGTATT  835
            ||||||||||||||||||||||||||||||||||||||||||||||||||||||||||||
Sbjct  778  TTCCTGAAAACGGACCCCAACAATAATGGGTTCTTTGGCGACGGTTCTCTCTTATGTATT  837

Query  836  CTTCGATCTATTGGCTTTAAACCGGAAAATTACACGAACTCTAACGTTAACAGGCTCCCG  895
            ||||||||||||||||||||||||||||||||||||||||||||||||||||||||||||
Sbjct  838  CTTCGATCTATTGGCTTTAAACCGGAAAATTACACGAACTCTAACGTTAACAGGCTCCCG  897

Query  896  ACCATGATTACGGATAGATACACGTTGGCTTCTAGATCCACAACATCCCGTTTACTTCAA  955
            ||||||||||||||||||||||||||||||||||||||||||||||||||||||||||||
Sbjct  898  ACCATGATTACGGATAGATACACGTTGGCTTCTAGATCCACAACATCCCGTTTACTTCAA  957

Query  956  AGTTATCTCAATAATTTTCACCCCTACTGCCCTATCGTGCACTCACCGACGCTAATGATG  1015
            ||||||||||||||||||||||||||||||||||||||||||||||||||||||||||||
Sbjct  958  AGTTATCTCAATAATTTTCACCCCTACTGCCCTATCGTGCACTCACCGACGCTAATGATG  1017

Query  1016 TTGTATAATAACCAGATTGAAATCGCGTCGAAGGATCAATGGCAAATCCTTTTTAACTGC  1075
            ||||||||||||||||||||||||||||||||||||||||||||||||||||||||||||
Sbjct  1018 TTGTATAATAACCAGATTGAAATCGCGTCGAAGGATCAATGGCAAATCCTTTTTAACTGC  1077

Query  1076 ATATTAGCCATTGGAGCCTGGTGTATAGAGGGGGAATCTACTGATATAGATGTTTTTTAC  1135
            ||||||||||||||||||||||||||||||||||||||||||||||||||||||||||||
Sbjct  1078 ATATTAGCCATTGGAGCCTGGTGTATAGAGGGGGAATCTACTGATATAGATGTTTTTTAC  1137

Query  1136 TATCAAAATGCTAAATCTCATTTGACGAGCAAGGTCTTCGAGTCAGGTTCCATAATTTTG  1195
            ||||||||||||||||||||||||||||||||||||||||||||||||||||||||||||
Sbjct  1138 TATCAAAATGCTAAATCTCATTTGACGAGCAAGGTCTTCGAGTCAGGTTCCATAATTTTG  1197

Query  1196 GTGACAGCCCTACATCTTCTGTCGCGATATACACAGTGGAGGCAGAAAACAAATACTAGC  1255
            ||||||||||||||||||||||||||||||||||||||||||||||||||||||||||||
Sbjct  1198 GTGACAGCCCTACATCTTCTGTCGCGATATACACAGTGGAGGCAGAAAACAAATACTAGC  1257

Query  1256 TATAATTTTCACAGCTTTTCCATAAGAATGGCCATATCATTGGGCTTGAATAGGGACCTC  1315
            ||||||||||||||||||||||||||||||||||||||||||||||||||||||||||||
Sbjct  1258 TATAATTTTCACAGCTTTTCCATAAGAATGGCCATATCATTGGGCTTGAATAGGGACCTC  1317

Query  1316 CCCTCGTCCTTCAGTGATAGCAGCATTCTGGAACAAAGACGCCGAATTTGGTGGTCTGTC  1375
            ||||||||||||||||||||||||||||||||||||||||||||||||||||||||||||
Sbjct  1318 CCCTCGTCCTTCAGTGATAGCAGCATTCTGGAACAAAGACGCCGAATTTGGTGGTCTGTC  1377

Query  1376 TACTCTTGGGAGATCCAATTGTCCCTGCTTTATGGTCGATCCATCCAGCTTTCTCAGAAT  1435
            ||||||||||||||||||||||||||||||||||||||||||||||||||||||||||||
Sbjct  1378 TACTCTTGGGAGATCCAATTGTCCCTGCTTTATGGTCGATCCATCCAGCTTTCTCAGAAT  1437

Query  1436 ACAATCTCCTTCCCTTCTTCTGTCGACGATGTGCAGCGTACCACAACAGGTCCCACCATA  1495
            ||||||||||||||||||||||||||||||||||||||||||||||||||||||||||||
Sbjct  1438 ACAATCTCCTTCCCTTCTTCTGTCGACGATGTGCAGCGTACCACAACAGGTCCCACCATA  1497

Query  1496 TATCATGGCATCATTGAAACAGCAAGGCTCTTACAAGTTTTCACAAAAATCTATGAACTA  1555
            ||||||||||||||||||||||||||||||||||||||||||||||||||||||||||||
Sbjct  1498 TATCATGGCATCATTGAAACAGCAAGGCTCTTACAAGTTTTCACAAAAATCTATGAACTA  1557
```

```
Query  1556 GACAAAACAGTAACTGCAGAAAAAAGTCCTATATGTGCAAAAAAATGCTTGATGATTTGT  1615
            ||||||||||||||||||||||||||||||||||||||||||||||||||||||||||||
Sbjct  1558 GACAAAACAGTAACTGCAGAAAAAAGTCCTATATGTGCAAAAAAATGCTTGATGATTTGT  1617

Query  1616 AATGAGATTGAGGAGGTTTCGAGACAGGCACCAAAGTTTTTACAAATGGATATTTCCACC  1675
            ||||||||||||||||||||||||||||||||||||||||||||||||||||||||||||
Sbjct  1618 AATGAGATTGAGGAGGTTTCGAGACAGGCACCAAAGTTTTTACAAATGGATATTTCCACC  1677

Query  1676 ACCGCTCTAACCAATTTGTTGAAGGAACACCCTTGGCTATCCTTTACAAGATTCGAACTG  1735
            ||||||||||||||||||||||||||||||||||||||||||||||||||||||||||||
Sbjct  1678 ACCGCTCTAACCAATTTGTTGAAGGAACACCCTTGGCTATCCTTTACAAGATTCGAACTG  1737

Query  1736 AAGTGGAAACAGTTGTCTCTTATCATTTATGTATTAAGAGATTTTTTCACTAATTTTACC  1795
            ||||||||||||||||||||||||||||||||||||||||||||||||||||||||||||
Sbjct  1738 AAGTGGAAACAGTTGTCTCTTATCATTTATGTATTAAGAGATTTTTTCACTAATTTTACC  1797

Query  1796 CAGAAAAAGTCACAACTAGAACAGGATCAAAATGATCATCAAAGTTATGAAGTTAAACGA  1855
            ||||||||||||||||||||||||||||||||||||||||||||||||||||||||||||
Sbjct  1798 CAGAAAAAGTCACAACTAGAACAGGATCAAAATGATCATCAAAGTTATGAAGTTAAACGA  1857

Query  1856 TGCTCCATCATGTTAAGCGATGCAGCACAAAGAACTGTTATGTCTGTAAGTAGCTATATG  1915
            ||||||||||||||||||||||||||||||||||||||||||||||||||||||||||||
Sbjct  1858 TGCTCCATCATGTTAAGCGATGCAGCACAAAGAACTGTTATGTCTGTAAGTAGCTATATG  1917

Query  1916 GACAATCATAATGTCACCCCATATTTTGCCTGGAATTGTTCTTATTACTTGTTCAATGCA  1975
            ||||||||||||||||||||||||||||||||||||||||||||||||||||||||||||
Sbjct  1918 GACAATCATAATGTCACCCCATATTTTGCCTGGAATTGTTCTTATTACTTGTTCAATGCA  1977

Query  1976 GTCCTAGTACCCATAAAGACTCTACTCTCAAACTCAAAATCGAATGCTGAGAATAACGAG  2035
            ||||||||||||||||||||||||||||||||||||||||||||||||||||||||||||
Sbjct  1978 GTCCTAGTACCCATAAAGACTCTACTCTCAAACTCAAAATCGAATGCTGAGAATAACGAG  2037

Query  2036 ACCGCACAATTATTACAACAAATTAACACTGTTCTGATGCTATTAAAAAAACTGGCCACT  2095
            ||||||||||||||||||||||||||||||||||||||||||||||||||||||||||||
Sbjct  2038 ACCGCACAATTATTACAACAAATTAACACTGTTCTGATGCTATTAAAAAAACTGGCCACT  2097

Query  2096 TTTAAAATCCAGACTTGTGAAAAATACATTCAAGTACTGGAAGAGGTATGTGCGCCGTTT  2155
            ||||||||||||||||||||||||||||||||||||||||||||||||||||||||||||
Sbjct  2098 TTTAAAATCCAGACTTGTGAAAAATACATTCAAGTACTGGAAGAGGTATGTGCGCCGTTT  2157

Query  2156 CTGTTATCACAGTGTGCAATCCCATTACCGCATATCAGTTATAACAATAGTAATGGTAGC  2215
            ||||||||||||||||||||||||||||||||||||||||||||||||||||||||||||
Sbjct  2158 CTGTTATCACAGTGTGCAATCCCATTACCGCATATCAGTTATAACAATAGTAATGGTAGC  2217

Query  2216 GCCATTAAAAATATTGTCGGTTCTGCAACTATCGCCCAATACCCTACTCTTCCGGAGGAA  2275
            ||||||||||||||||||||||||||||||||||||||||||||||||||||||||||||
Sbjct  2218 GCCATTAAAAATATTGTCGGTTCTGCAACTATCGCCCAATACCCTACTCTTCCGGAGGAA  2277

Query  2276 AATGTCAACAATATCAGTGTTAAATATGTTTCTCCTGGCTCAGTAGGGCCTTCACCTGTG  2335
            ||||||||||||||||||||||||||||||||||||||||||||||||||||||||||||
Sbjct  2278 AATGTCAACAATATCAGTGTTAAATATGTTTCTCCTGGCTCAGTAGGGCCTTCACCTGTG  2337

Query  2336 CCATTGAAATCAGGAGCAAGTTTCAGTGATCTAGTCAAGCTGTTATCTAACCGTCCACCC  2395
            ||||||||||||||||||||||||||||||||||||||||||||||||||||||||||||
Sbjct  2338 CCATTGAAATCAGGAGCAAGTTTCAGTGATCTAGTCAAGCTGTTATCTAACCGTCCACCC  2397
```

```
Query  2396  TCTCGTAACTCTCCAGTGACAATACCAAGAAGCACACCTTCGCATCGCTCAGTCACGCCT  2455
             ||||||||||||||||||||||||||||||||||||||||||||||||||||||||||||
Sbjct  2398  TCTCGTAACTCTCCAGTGACAATACCAAGAAGCACACCTTCGCATCGCTCAGTCACGCCT  2457

Query  2456  TTTCTAGGGCAACAGCAACAGCTGCAATCATTAGTGCCACTGACCCCGTCTGCTTTGTTT  2515
             ||||||||||||||||||||||||||||||||||||||||||||||||||||||||||||
Sbjct  2458  TTTCTAGGGCAACAGCAACAGCTGCAATCATTAGTGCCACTGACCCCGTCTGCTTTGTTT  2517

Query  2516  GGTGGCGCCAATTTTAATCAAAGTGGGAATATTGCTGATAGCTCATTGTCCTTCACTTTC  2575
             ||||||||||||||||||||||||||||||||||||||||||||||||||||||||||||
Sbjct  2518  GGTGGCGCCAATTTTAATCAAAGTGGGAATATTGCTGATAGCTCATTGTCCTTCACTTTC  2577

Query  2576  ACTAACAGTAGCAACGGTCCGAACCTCATAACAACTCAAACAAATTCTCAAGCGCTTTCA  2635
             ||||||||||||||||||||||||||||||||||||||||||||||||||||||||||||
Sbjct  2578  ACTAACAGTAGCAACGGTCCGAACCTCATAACAACTCAAACAAATTCTCAAGCGCTTTCA  2637

Query  2636  CAACCAATTGCCTCCTCTAACGTTCATGATAACTTCATGAATAATGAAATCACGGCTAGT  2695
             ||||||||||||||||||||||||||||||||||||||||||||||||||||||||||||
Sbjct  2638  CAACCAATTGCCTCCTCTAACGTTCATGATAACTTCATGAATAATGAAATCACGGCTAGT  2697

Query  2696  AAAATTGATGATGGTAATAATTCAAAACCACTGTCACCTGGTTGGACGGACCAAACTGCG  2755
             ||||||||||||||||||||||||||||||||||||||||||||||||||||||||||||
Sbjct  2698  AAAATTGATGATGGTAATAATTCAAAACCACTGTCACCTGGTTGGACGGACCAAACTGCG  2757

Query  2756  TATAACGCGTTTGGAATCACTACAGGGATGTTTAATACCACTACAATGGATGATGTATAT  2815
             ||||||||||||||||||||||||||||||||||||||||||||||||||||||||||||
Sbjct  2758  TATAACGCGTTTGGAATCACTACAGGGATGTTTAATACCACTACAATGGATGATGTATAT  2817

Query  2816  AACTATCTATTCGATGATGAAGATACCCCACCAAACCCAAAAAAAGAGTAAAATGAATCG  2875
             ||||||||||||||||||||||||||||||||||||||||||||||||||||||||||||
Sbjct  2818  AACTATCTATTCGATGATGAAGATACCCCACCAAACCCAAAAAAAGAGTAAAATGAATCG  2877

Query  2876  TAGATACTGAAAAACCCCGCAAGTTCACTTCAACTGTGCATCGTGCACCATCTCAATTTC  2935
             ||||||||||||||||||||||||||||||||||||||||||||||||||||||||||||
Sbjct  2878  TAGATACTGAAAAACCCCGCAAGTTCACTTCAACTGTGCATCGTGCACCATCTCAATTTC  2937

Query  2936  TTTCATTTATACATCGTTTTGCCTTCTTTTATGTAACTATACTCCTCTAAGTTTCAATCT  2995
             ||||||||||||||||||||||||||||||||||||||||||||||||||||||||||||
Sbjct  2938  TTTCATTTATACATCGTTTTGCCTTCTTTTATGTAACTATACTCCTCTAAGTTTCAATCT  2997

Query  2996  TGGCCATGTAACCTCTGATCTATAGAATTTTTTAAATGACTAGAATTAATGCCCATCTTT  3055
             ||||||||||||||||||||||||||||||||||||||||||||||||||||||||||||
Sbjct  2998  TGGCCATGTAACCTCTGATCTATAGAATTTTTTAAATGACTAGAATTAATGCCCATCTTT  3057

Query  3056  TTTTTGGACCTAAATTCTTCATGAAAATATATTACGAGGGCTTATTCAGAAGCTTATCGA  3115
             ||||||||||||||||||||||||||||||||||||||||||||||||||||||||||||
Sbjct  3058  TTTTTGGACCTAAATTCTTCATGAAAATATATTACGAGGGCTTATTCAGAAGCTTATCGA  3117

Query  3116  TACCGTCGACTAAAGCCAAATAGAAAATTATTCAGTTCCTGGCTTAAGTTTTTAAAAGTG  3175
             |||||||||||||||||||||||||| ||||||||||| |||||||||||||||||||||
Sbjct  3118  TACCGTCGACTAAAGCCAAATAGAAA-TTATTCAGTTC-TGGCTTAAGTTTTTAAAAGTG  3175

Query  3176  ATATTATTTATTTGGTTGTAACCAACCAAAAGAATGTAAATAACTAATACATAATTATGT  3235
             ||||||||||||||||||||||||||||||||||||||||||||||||||||||||||||
Sbjct  3176  ATATTATTTATTTGGTTGTAACCAACCAAAAGAATGTAAATAACTAATACATAATTATGT  3235
```

```
Query  3236  TAGTTTTAAGTTAGCAACAAATTGATTTTAGCTATATTAGCTACTTGGTTAATAAATAGA  3295
             ||||||||||||||||||||||||||||||||||||||||||||||||||||||||||||
Sbjct  3236  TAGTTTTAAGTTAGCAACAAATTGATTTTAGCTATATTAGCTACTTGGTTAATAAATAGA  3295

Query  3296  ATATATTTATTTAAAGATAATTGCGTTTTTATTGTCAGGGAGTGAGTTTGCTTAAAAACT  3355
             |||||||||||||||||||||| |||||||||||||||||||||||||||||||||||||
Sbjct  3296  ATATATTTATTTAAAGATAATT-CGTTTTTATTGTCAGGGAGTGAGTTTGCTTAAAAACT  3354

Query  3356  CGTTTAGATCCACTAGTTCTAGAGCGGCCGCAGCTTGATATCGAATTCCTGCAGCCCGGG  3415
             ||||||||||||||||||||||||||||||||||||||||||||||||||||||||||||
Sbjct  3355  CGTTTAGATCCACTAGTTCTAGAGCGGCCGCAGCTTGATATCGAATTCCTGCAGCCCGGG  3414

Query  3416  GGATCCACTAGTTCTAGAGCGGCCCCCCGTTATTCTCTATTCGTTTTGTGACTCTCCCTC  3475
             ||||||||||||||||||||||||||||||||||||||||||||||||||||||||||||
Sbjct  3415  GGATCCACTAGTTCTAGAGCGGCCCCCCGTTATTCTCTATTCGTTTTGTGACTCTCCCTC  3474

Query  3476  TCTGTACTATTGCTCTCTCACTCTGTCGCACAGTAAACGGCACACTATTCTCGTTGCTTC  3535
             ||||||||||||||||||||||||||||||||||||||||||| ||||||||||||||||
Sbjct  3475  TCTGTACTATTGCTCTCTCACTCTGTCGCACAGTAAACGGCACTCTATTCTCGTTGCTTC  3534

Query  3536  GAGAGAGCGCGCCTCGAATGTTCGCGAAAAGAGCGCCGGAGTATAAATAGAGGCGCTTCG  3595
             ||||||||||||||||||||||||||||||||||||||||||||||||||||||||||||
Sbjct  3535  GAGAGAGCGCGCCTCGAATGTTCGCGAAAAGAGCGCCGGAGTATAAATAGAGGCGCTTCG  3594

Query  3596  TCGACGGAGCGTCAATTCAATTCAAACAAGCAAAGTGAACACATCGCGAAGCGTAAGCTG  3655
             ||||||||||||||||||||||||||||||||||||||||||||||||||||  ||||||
Sbjct  3595  TCGACGGAGCGTCAATTCAATTCAAACAAGCAAAGTGAACACATCGCGAAGC--AAGCTG  3652

Query  3656  AGCAAACAAACAAGCGCAGCTGAACAAGCTAAACAATCTGCAATAAAGTGCAAGTTAAAG  3715
             |||||||||||||||||||| |||||||||||||||||||||||||||||||||||||||
Sbjct  3653  AGCAAACAAACAAGCGCAGC-GAACAAGCTAAACAATCTGCAATAAAGTGCAAGTTAAAG  3711

Query  3716  TGAATCAATTAAAAGTAACCAACAACCAAGTAATTAAACTAAAAACTGCAACTACTGAAA  3775
             ||||||||||||||||||||||||||||||||||||||||||||||||||||||||||||
Sbjct  3712  TGAATCAATTAAAAGTAACCAACAACCAAGTAATTAAACTAAAAACTGCAACTACTGAAA  3771

Query  3776  TCAACCAAGAAGTCATTATTGAAGACAAGAAGAGAACTCTGAATAGGTCGATAGCGTCAA  3835
             ||||||||||||||||||||||||||||||||||||||||||||||||||||||||||||
Sbjct  3772  TCAACCAAGAAGTCATTATTGAAGACAAGAAGAGAACTCTGAATAGGTCGATAGCGTCAA  3831

Query  3836  TGTCCGCCTTCAGTTGCACTTTGTCAGCGGTTTCGTGACGAAGCTCCAAGCGGTTTACGC  3895
             ||||||||||||||||||||||||||||||||||||||||||||||||||||||||||||
Sbjct  3832  TGTCCGCCTTCAGTTGCACTTTGTCAGCGGTTTCGTGACGAAGCTCCAAGCGGTTTACGC  3891

Query  3896  CATCAATTAAACACAAAGTGCTGTGCCAAAACTCCTCTCGCTTCTTATTTTTGTTTGTTT  3955
             ||||||||||||||||||||||||||||||||||||||||||||||||||||||||||||
Sbjct  3892  CATCAATTAAACACAAAGTGCTGTGCCAAAACTCCTCTCGCTTCTTATTTTTGTTTGTTT  3951

Query  3956  TTTGAGTGATTGGGGTGGTGATTGGTTTTGGGTGGGTAAGCAGGGGAAAGTGTGAAAAAT  4015
             ||||||||||||||||||||||||||||||||||||||||||||||||||||||||||||
Sbjct  3952  TTTGAGTGATTGGGGTGGTGATTGGTTTTGGGTGGGTAAGCAGGGGAAAGTGTGAAAAAT  4011

Query  4016  CCCGGCAATGGGCCAAGAGGATCAGGAGCTATTAATTCGCGGAGGCAGCAAACACCCATC  4075
             ||||||||||||||||||||||||||||||||||||||||||||||||||||||||||||
Sbjct  4012  CCCGGCAATGGGCCAAGAGGATCAGGAGCTATTAATTCGCGGAGGCAGCAAACACCCATC  4071
```

```
Query  4076  TGCCGAGCATCTGAACAATGTGAGTAGTACATGTGCATACATCTTAAGTTCACTTGATCT  4135
             ||||||||||||||||||||||||||||||||||||||||||||||||||||||||||||
Sbjct  4072  TGCCGAGCATCTGAACAATGTGAGTAGTACATGTGCATACATCTTAAGTTCACTTGATCT  4131

Query  4136  ATAGGAACTGCGATTGCAACATCAAATTGTCTGCGGCGTGAGAACTGCGACCCACAAAAA  4195
             ||||||||||||||||||||||||||||||||||||||||||||||||||||||||||||
Sbjct  4132  ATAGGAACTGCGATTGCAACATCAAATTGTCTGCGGCGTGAGAACTGCGACCCACAAAAA  4191

Query  4196  TCCCAAACCGCAATCGCACAAACAAATAGTGACACGAAACAGATTATTCTGGTAGCTGTG  4255
             ||||||||||||||||||||||||||||||||||||||||||||||||||||||||||||
Sbjct  4192  TCCCAAACCGCAATCGCACAAACAAATAGTGACACGAAACAGATTATTCTGGTAGCTGTG  4251

Query  4256  CTCGCTATATAAGACAATTTTTAAGATCATATCATGATCAAGACATCTAAAGGCATTCAT  4315
             ||||||||||||||||||||||||||||||||||||||||||||||||||||||||||||
Sbjct  4252  CTCGCTATATAAGACAATTTTTAAGATCATATCATGATCAAGACATCTAAAGGCATTCAT  4311

Query  4316  TTTCGACTACATTCTTTTTTACAAAAAATATAACAACCAGATATTTTAAGCT-CGACTCT  4374
             |||||||||||||||||||||||||||||||||||||||||||||||||||| |||||||
Sbjct  4312  TTTCGACTACATTCTTTTTTACAAAAAATATAACAACCAGATATTTTAAGCTTCGACTCT  4371

Query  4375  AGCTAGATGCACAAAAAATAAATAAAAGTATAAACCTACTTCGTAGGATACTTCGTTTTG  4434
             ||||||||||||||||||||||||||||||||||||||||||||||||||||||||||||
Sbjct  4372  AGCTAGATGCACAAAAAATAAATAAAAGTATAAACCTACTTCGTAGGATACTTCGTTTTG  4431

Query  4435  TTCGGGGTTAGATGAGCATAACGCTTGTAGTTGATATTTGAGATCCCCTATCATTGCAGG  4494
             ||||||||||||||||||||||||||||||||||||||||||||||||||||||||||||
Sbjct  4432  TTCGGGGTTAGATGAGCATAACGCTTGTAGTTGATATTTGAGATCCCCTATCATTGCAGG  4491

Query  4495  GTGACAGCGGAGCGGCTTCGCAGAGCTGCATTAACCAGGGCTTCGGGCAGGCCAAAAACT  4554
             ||||||||||| || |||||||||||||||||||||||||||||||||||||||||||||
Sbjct  4492  GTGACAGCGGA-CG-CTTCGCAGAGCTGCATTAACCAGGGCTTCGGGCAGGCCAAAAACT  4549

Query  4555  ACGGCACGCTCCTGCCACCCAGTCCGCCGGAGGACTCCGGTTCAGGGAGCGGCCAACTAG  4614
             ||||||||||||||||||||||||||||||||||||||||||||||||||||||||||||
Sbjct  4550  ACGGCACGCTCCTGCCACCCAGTCCGCCGGAGGACTCCGGTTCAGGGAGCGGCCAACTAG  4609

Query  4615  CCGAGAACCTCACCTATGCCTGGCACAATATGGACATCTTTGGGGCGGTCAATCAGCCGG  4674
             ||||||||||||||||||||||||||||||||||||||||||||||||||||||||||||
Sbjct  4610  CCGAGAACCTCACCTATGCCTGGCACAATATGGACATCTTTGGGGCGGTCAATCAGCCGG  4669

Query  4675  GCTCCGGATGGCGGCAGCTGGTCAACCGGACACGCGGACTATTCTGCAACGAGCGACACA  4734
             ||||||||||||||||||||||||||||||||||||||||||||||||||||||||||||
Sbjct  4670  GCTCCGGATGGCGGCAGCTGGTCAACCGGACACGCGGACTATTCTGCAACGAGCGACACA  4729

Query  4735  TACCGGCGCCCAGGAAACATTTGCTCAAGAACGGTGAGTTTCTATTCGCAGTCGGCTGAT  4794
             ||||||||||||||||||||||||||||||||||||||||||||||||||||||||||||
Sbjct  4730  TACCGGCGCCCAGGAAACATTTGCTCAAGAACGGTGAGTTTCTATTCGCAGTCGGCTGAT  4789

Query  4795  CTGTGTGAAATCTTAATAAAGGGTCCAATTACCAATTTGAAACTCAGTTTGCGGCGTGGC  4854
             ||||||||||||||||||||||||||||||||||||||||||||||||||||||||||||
Sbjct  4790  CTGTGTGAAATCTTAATAAAGGGTCCAATTACCAATTTGAAACTCAGTTTGCGGCGTGGC  4849

Query  4855  CTATCCGGGCGAACTTTTGGCCGTGATGGGCAGTTCCGGTGCCGGAAAGACGACCCTGCT  4914
             ||||||||||||||||||||||||||||||||||||||||||||||||||||||||||||
Sbjct  4850  CTATCCGGGCGAACTTTTGGCCGTGATGGGCAGTTCCGGTGCCGGAAAGACGACCCTGCT  4909
```

```
Query  4915 GAATGCCCTTGCCTTTCGATCGCCGCAGGGCATCCAAGTATCGCCATCCGGGATGCGACT  4974
            ||||||||||||||||||||||||||||||||||||||||||||||||||||||||||||
Sbjct  4910 GAATGCCCTTGCCTTTCGATCGCCGCAGGGCATCCAAGTATCGCCATCCGGGATGCGACT  4969

Query  4975 GCTCAATGGCCAACCTGTGGACGCCAAGGAGATGCAGGCCAGGTGCGCCTATGTCCAGCA  5034
            ||||||||||||||||||||||||||||||||||||||||||||||||||||||||||||
Sbjct  4970 GCTCAATGGCCAACCTGTGGACGCCAAGGAGATGCAGGCCAGGTGCGCCTATGTCCAGCA  5029

Query  5035 GGATGACCTCTTTATCGGCTCCCTAACGGCCAGGGAACACCTGATTTTCCAGGCCATGGT  5094
            ||||||||||||||||||||||||||||||||||||||||||||||||||||||||||||
Sbjct  5030 GGATGACCTCTTTATCGGCTCCCTAACGGCCAGGGAACACCTGATTTTCCAGGCCATGGT  5089

Query  5095 GCGGATGCCACGACATCTGACCTATCGGCAGCGAGTGGCCCGCGTGGATCAGGTGATCCA  5154
            ||||||||||||||||||||||||||||||||||||||||||||||||||||||||||||
Sbjct  5090 GCGGATGCCACGACATCTGACCTATCGGCAGCGAGTGGCCCGCGTGGATCAGGTGATCCA  5149

Query  5155 GGAGCTTTCGCTCAGCAAATGTCAGCACACGATCATCGGTGTGCCCGGCAGGGTGAAAGG  5214
            ||||||||||||||||||||||||||||||||||||||||||||||||||||||||||||
Sbjct  5150 GGAGCTTTCGCTCAGCAAATGTCAGCACACGATCATCGGTGTGCCCGGCAGGGTGAAAGG  5209

Query  5215 TCTGTCCGGCGGAGAAAGGAAGCGTCTGGCATTCGCCTCCGAGGCACTAACCGATCCGCC  5274
            ||||||||||||||||||||||||||||||||||||||||||||||||||||||||||||
Sbjct  5210 TCTGTCCGGCGGAGAAAGGAAGCGTCTGGCATTCGCCTCCGAGGCACTAACCGATCCGCC  5269

Query  5275 GCTTCTGATCTGCGATGAGCCCACCTCCGGACTGGACTCATTTACCGCCCACAGCGTCGT  5334
            ||||||||||||||||||||||||||||||||||||||||||||||||||||||||||||
Sbjct  5270 GCTTCTGATCTGCGATGAGCCCACCTCCGGACTGGACTCATTTACCGCCCACAGCGTCGT  5329

Query  5335 CCAGGTGCTGAAGAAGCTGTCGCAGAAGGGCAAGACCGTCATCCTGACCATTCATCAGCC  5394
            ||||||||||||||||||||||||||||||||||||||||||||||||||||||||||||
Sbjct  5330 CCAGGTGCTGAAGAAGCTGTCGCAGAAGGGCAAGACCGTCATCCTGACCATTCATCAGCC  5389

Query  5395 GTCTTCCGAGCTGTTTGAGCTCTTTGACAAGATCCTTCTGATGGCCGAGGGCAGGGTAGC  5454
            ||||||||||||||||||||||||||||||||||||||||||||||||||||||||||||
Sbjct  5390 GTCTTCCGAGCTGTTTGAGCTCTTTGACAAGATCCTTCTGATGGCCGAGGGCAGGGTAGC  5449

Query  5455 TTTCTTGGGCACTCCCAGCGAAGCCGTCGACTTCTTTTCCTAGTGAGTTCGATGTGTTTA  5514
            ||||||||||||||||||||||||||||||||||||||||||||||||||||||||||||
Sbjct  5450 TTTCTTGGGCACTCCCAGCGAAGCCGTCGACTTCTTTTCCTAGTGAGTTCGATGTGTTTA  5509

Query  5515 TTAAGGGTATCTAGCATTACATTACATCTCAACTCCTATCCAGCGTGGGTGCCCAGTGTC  5574
            ||||||||||||||||||||||||||||||||||||||||||||||||||||||||||||
Sbjct  5510 TTAAGGGTATCTAGCATTACATTACATCTCAACTCCTATCCAGCGTGGGTGCCCAGTGTC  5569

Query  5575 CTACCAACTACAATCCGGCGGACTTTTACGTACAGGTGTTGGCCGTTGTGCCCGGACGGG  5634
            ||||||||||||||||||||||||||||||||||||||||||||||||||||||||||||
Sbjct  5570 CTACCAACTACAATCCGGCGGACTTTTACGTACAGGTGTTGGCCGTTGTGCCCGGACGGG  5629

Query  5635 AGATCGAGTCCCGTGATCGGATCGCCAAGATATGCGACAATTTTGCTATTAGCAAAGTAG  5694
            ||||||||||||||||||||||||||||||||||||||||||||||||||||||||||||
Sbjct  5630 AGATCGAGTCCCGTGATCGGATCGCCAAGATATGCGACAATTTTGCTATTAGCAAAGTAG  5689

Query  5695 CCCGGGATATGGAGCAGTTGTTGGCCACCAAAAATTTGGAGAAGCCACTGGAGCAGCCGG  5754
            ||||||||||||||||||||||||||||||||||||||||||||||||||||||||||||
Sbjct  5690 CCCGGGATATGGAGCAGTTGTTGGCCACCAAAAATTTGGAGAAGCCACTGGAGCAGCCGG  5749
```

```
Query  5755  AGAATGGGTACACCTACAAGGCCACCTGGTTCATGCAGTTCCGGGCGGTCCTGTGGCGAT  5814
             ||||||||||||||||||||||||||||||||||||||||||||||||||||||||||||
Sbjct  5750  AGAATGGGTACACCTACAAGGCCACCTGGTTCATGCAGTTCCGGGCGGTCCTGTGGCGAT  5809

Query  5815  CCTGGCTGTCGGTGCTCAAGGAACCACTCCTCGTAAAAGTGCGACTTATTCAGACAACGG  5874
             ||||||||||||||||||||||||||||||||||||||||||||||||||||||||||||
Sbjct  5810  CCTGGCTGTCGGTGCTCAAGGAACCACTCCTCGTAAAAGTGCGACTTATTCAGACAACGG  5869

Query  5875  TGAGTGGTTCCAGTGGAAACAAATGATATAACGCTTACAATTCTTGGAAACAAATTCGCT  5934
             ||||||||||||||||||||||||||||||||||||||||||||||||||||||||||||
Sbjct  5870  TGAGTGGTTCCAGTGGAAACAAATGATATAACGCTTACAATTCTTGGAAACAAATTCGCT  5929

Query  5935  AGATTTTAGTTAGAATTGCCTGATTCCACACCCTTCTTAGTTTTTTTCAATGAGATGTAT  5994
             ||||||||||||||||||||||||||||||||||||||||||||||||||||||||||||
Sbjct  5930  AGATTTTAGTTAGAATTGCCTGATTCCACACCCTTCTTAGTTTTTTTCAATGAGATGTAT  5989

Query  5995  AGTTTATAGTTTTGCAGAAGATAAATAAATTTCATTTAACTCGCGAACATGTTGAAGATA  6054
             ||||||||||||||||||| ||||||||||||||||||||||||||||||||||||||||
Sbjct  5990  AGTTTATAGTTTTGCAGAAAATAAATAAATTTCATTTAACTCGCGAACATGTTGAAGATA  6049

Query  6055  TGAATATTAATGAGATGCGAGTAACATTTTAATTTGCAGATGGTTGCCATCTTGATTGGC  6114
             ||||||||||||||||||||||||||||||||||||||||||||||||||||||||||||
Sbjct  6050  TGAATATTAATGAGATGCGAGTAACATTTTAATTTGCAGATGGTTGCCATCTTGATTGGC  6109

Query  6115  CTCATCTTTTTGGGCCAACAACTCACGCAAGTGGGCGTGATGAATATCAACGGAGCCATC  6174
             ||||||||||||||||||||||||||||||||||||||||||||||||||||||||||||
Sbjct  6110  CTCATCTTTTTGGGCCAACAACTCACGCAAGTGGGCGTGATGAATATCAACGGAGCCATC  6169

Query  6175  TTCCTCTTCCTGACCAACATGACCTTTCAAAACGTCTTTGCCACGATAAATGTAAGTCTT  6234
             ||||||||||||||||||||||||||||||||||||||||||||||||||||||||||||
Sbjct  6170  TTCCTCTTCCTGACCAACATGACCTTTCAAAACGTCTTTGCCACGATAAATGTAAGTCTT  6229

Query  6235  GTTTAGAATACATTTGCATATTAATAATTTACTAACTTTCTAATGAATCGATTCGATTTA  6294
             ||||||||||||||||||||||||||||||||||||||||||||||||||||||||||||
Sbjct  6230  GTTTAGAATACATTTGCATATTAATAATTTACTAACTTTCTAATGAATCGATTCGATTTA  6289

Query  6295  GGTGTTCACCTCAGAGCTGCCAGTTTTTATGAGGGAGGCCCGAAGTCGACTTTATCGCTG  6354
             ||||||||||||||||||||||||||||||||||||||||||||||||||||||||||||
Sbjct  6290  GGTGTTCACCTCAGAGCTGCCAGTTTTTATGAGGGAGGCCCGAAGTCGACTTTATCGCTG  6349

Query  6355  TGACACATACTTTCTGGGCAAAACGATTGCCGAATTACCGCTTTTTCTCACAGTGCCACT  6414
             ||||||||||||||||||||||||||||||||||||||||||||||||||||||||||||
Sbjct  6350  TGACACATACTTTCTGGGCAAAACGATTGCCGAATTACCGCTTTTTCTCACAGTGCCACT  6409

Query  6415  GGTCTTCACGGCGATTGCCTATCCGATGATCGGACTGCGGGCCGGAGTGCTGCACTTCTT  6474
             ||||||||||||||||||||||||||||||||||||||||||||||||||||||||||||
Sbjct  6410  GGTCTTCACGGCGATTGCCTATCCGATGATCGGACTGCGGGCCGGAGTGCTGCACTTCTT  6469

Query  6475  CAACTGCCTGGCGCTGGTCACTCTGGTGGCCAATGTGTCAACGTCCTTCGGATATCTAAT  6534
             ||||||||||||||||||||||||||||||||||||||||||||||||||||||||||||
Sbjct  6470  CAACTGCCTGGCGCTGGTCACTCTGGTGGCCAATGTGTCAACGTCCTTCGGATATCTAAT  6529

Query  6535  ATCCTGCGCCAGCTCCTCGACCTCGATGGCGCTGTCTGTGGGTCCGCCGGTTATCATACC  6594
             ||||||||||||||||||||||||||||||||||||||||||||||||||||||||||||
Sbjct  6530  ATCCTGCGCCAGCTCCTCGACCTCGATGGCGCTGTCTGTGGGTCCGCCGGTTATCATACC  6589
```

```
Query  6595  ATTCCTGCTCTTTGGCGGCTTCTTCTTGAACTCGGGCTCGGTGCCAGTATACCTCAAATG  6654
             ||||||||||||||||||||||||||||||||||||||||||||||||||||||||||||
Sbjct  6590  ATTCCTGCTCTTTGGCGGCTTCTTCTTGAACTCGGGCTCGGTGCCAGTATACCTCAAATG  6649

Query  6655  GTTGTCGTACCTCTCATGGTTCCGTTACGCCAACGAGGGTCTGCTGATTAACCAATGGGC  6714
             ||||||||||||||||||||||||||||||||||||||||||||||||||||||||||||
Sbjct  6650  GTTGTCGTACCTCTCATGGTTCCGTTACGCCAACGAGGGTCTGCTGATTAACCAATGGGC  6709

Query  6715  GGACGTGGAGCCGGGCGAAATTAGCTGCACATCGTCGAACACCACGTGCCCCAGTTCGGG  6774
             ||||||||||||||||||||||||||||||||||||||||||||||||||||||||||||
Sbjct  6710  GGACGTGGAGCCGGGCGAAATTAGCTGCACATCGTCGAACACCACGTGCCCCAGTTCGGG  6769

Query  6775  CAAGGTCATCCTGGAGACGCTTAACTTCTCCGCCGCCGATCTGCCGCTGGACTACGTGGG  6834
             ||||||||||||||||||||||||||||||||||||||||||||||||||||||||||||
Sbjct  6770  CAAGGTCATCCTGGAGACGCTTAACTTCTCCGCCGCCGATCTGCCGCTGGACTACGTGGG  6829

Query  6835  TCTGGCCATTCTCATCGTGAGCTTCCGGGTGCTCGCATATCTGGCTCTAAGACTTCGGGC  6894
             ||||||||||||||||||||||||||||||||||||||||||||||||||||||||||||
Sbjct  6830  TCTGGCCATTCTCATCGTGAGCTTCCGGGTGCTCGCATATCTGGCTCTAAGACTTCGGGC  6889

Query  6895  CCGACGCAAGGAGTAGCCGACATATATCCGAAATAACTGCTTGTTTTTTTTTTTACCATT  6954
             ||||||||||||||||||||||||||||||||||||||||||||||||||||||||||||
Sbjct  6890  CCGACGCAAGGAGTAGCCGACATATATCCGAAATAACTGCTTGTTTTTTTTTTTACCATT  6949

Query  6955  ATTACCATCGTGTTTACTGTTTATTGCCCCCTCAAAAAGCTAATGTAATTATATTTGTGC  7014
             ||||||||||||||||||||||||||||||||||||||||||||||||||||||||||||
Sbjct  6950  ATTACCATCGTGTTTACTGTTTATTGCCCCCTCAAAAAGCTAATGTAATTATATTTGTGC  7009

Query  7015  CAATAAAAACAAGATATGACCTATAGAATACAAGTATTTCCCCTTCGAACATCCCCACAA  7074
             ||||||||||||||||||||||||||||||||||||||||||||||||||||||||||||
Sbjct  7010  CAATAAAAACAAGATATGACCTATAGAATACAAGTATTTCCCCTTCGAACATCCCCACAA  7069

Query  7075  GTAGACTTTGGATTTGTCTTCTAACCAAAAGACTTACACACCTGCATACCTTACATCAAA  7134
             ||||||||||||||||||||||||||||||||||||||||||||||||||||||||||||
Sbjct  7070  GTAGACTTTGGATTTGTCTTCTAACCAAAAGACTTACACACCTGCATACCTTACATCAAA  7129

Query  7135  AACTCGTTTATCGCTACATAAAACACCGGGATATATTTTTTATATACATACTTTTCAAAT  7194
             ||||||||||||||||||||||||||||||||||||||||||||||||||||||||||||
Sbjct  7130  AACTCGTTTATCGCTACATAAAACACCGGGATATATTTTTTATATACATACTTTTCAAAT  7189

Query  7195  CGCGCGCCCTCTTCATAATTCACCTCCACCACACCACGTTTCGTAGTTGCTCTTTCGCTG  7254
             ||||||||||||||||||||||||||||||||||||||||||||||||||||||||||||
Sbjct  7190  CGCGCGCCCTCTTCATAATTCACCTCCACCACACCACGTTTCGTAGTTGCTCTTTCGCTG  7249

Query  7255  TCTCCCACCCGCTCTCCGCAACACATTCACCTTTTGTTCGACGACCTTGGAGCGACTGTC  7314
             ||||||||||||||||||||||||||||||||||||||||||||||||||||||||||||
Sbjct  7250  TCTCCCACCCGCTCTCCGCAACACATTCACCTTTTGTTCGACGACCTTGGAGCGACTGTC  7309

Query  7315  GTTAGTTCCGCGCGATTCGGTTCGCTCAAATGGTTCCGAGTGGTTCATTTCGTCTCAATA  7374
             ||||||||||||||||||||||||||||||||||||||||||||||||||||||||||||
Sbjct  7310  GTTAGTTCCGCGCGATTCGGTTCGCTCAAATGGTTCCGAGTGGTTCATTTCGTCTCAATA  7369

Query  7375  GAAATTAGTAATAAATATTTGTATGTACAATTTATTTGCTCCAATATATTTGTATATATT  7434
             ||||||||||||||||||||||||||||||||||||||||||||||||||||||||||||
Sbjct  7370  GAAATTAGTAATAAATATTTGTATGTACAATTTATTTGCTCCAATATATTTGTATATATT  7429
```

```
Query  7435  TCCCTCACAGCTATATTTATTCTAATTTAATATTATGACTTTTTAAGGTAATTTTTTGTG  7494
             ||||||||||||||||||||||||||||||||||||||||||||||||||||||||||||
Sbjct  7430  TCCCTCACAGCTATATTTATTCTAATTTAATATTATGACTTTTTAAGGTAATTTTTTGTG  7489

Query  7495  ACCTGTTCGGAGTGATTAGCGTTACAATTTGAACTGAAAGTGACATCCAGTGTTTGTTCC  7554
             ||||||||||||||||||||||||||||||||||||||||||||||||||||||||||||
Sbjct  7490  ACCTGTTCGGAGTGATTAGCGTTACAATTTGAACTGAAAGTGACATCCAGTGTTTGTTCC  7549

Query  7555  TTGTGTAGATGCATCTCAAAAAAATGGTGGGCATAATAGTGTTGTTTATATATATCAAAA  7614
             ||||||||||||||||||||||||||||||||||||||||||||||||||||||||||||
Sbjct  7550  TTGTGTAGATGCATCTCAAAAAAATGGTGGGCATAATAGTGTTGTTTATATATATCAAAA  7609

Query  7615  ATAACAACTATAATAATAAGAATACATTTAATTTAGAAAATGCTTGGATTTCACTGGAAC  7674
             ||||||||||||||||||||||||||||||||||||||||||||||||||||||||||||
Sbjct  7610  ATAACAACTATAATAATAAGAATACATTTAATTTAGAAAATGCTTGGATTTCACTGGAAC  7669

Query  7675  TAGTTCTCTCTCTCTCTCTCTTATCTATCGCTACTTGGTTGGCGCGCTCTCGCGCTCTCT  7734
             ||||||||||||||||||||||||||||||||||||||||||||||||||||||||||||
Sbjct  7670  TAGTTCTCTCTCTCTCTCTCTTATCTATCGCTACTTGGTTGGCGCGCTCTCGCGCTCTCT  7729

Query  7735  TTGTGTGCGTGTGGGCAGTGTGTTTTTTGTTGTTTTGCGCTTTATGTGTTGTATTTTGTG  7794
             ||||||||||||||||||||||||||||||||||||||||||||||||||||||||||||
Sbjct  7730  TTGTGTGCGTGTGGGCAGTGTGTTTTTTGTTGTTTTGCGCTTTATGTGTTGTATTTTGTG  7789

Query  7795  TGTTTGGCCGAAGTATTTAAAACAAAAGTGCAGCGGAAATAGTTAATAACAAAATATTAG  7854
             ||||||||||||||||||||||||||||||||||||||||||||||||||||||||||||
Sbjct  7790  TGTTTGGCCGAAGTATTTAAAACAAAAGTGCAGCGGAAATAGTTAATAACAAAATATTAG  7849

Query  7855  TCGACGGTAGCGGTCCGGTTGTTTTCGTGCTCATCGCGAGTACGTATTTATTTTTAAAAA  7914
             |||||
Sbjct  7850  TCGACNNNNNNNNNNNNNNNNNNNNNNNNNNNNNNNNNNNNNNNNNNNNNNNNNNNNNNN  7909

Query  7915  ATAATAAACGCGGCAAACACAATCACACAAATGTGCAGGCACAGTGGGACAAAGTGCGCT  7974

Sbjct  7910  NNNNNNNNNNNNNNNNNNNNNNNNNNNNNNNNNNNNNNNNNNNNNNNNNNNNNNNNNNNN  7969

Query  7975  AAATTTGGATATGTAATAAATCCAAAACCAAAAAGAAAACAAAAAACGGTAAAATATTTT  8034

Sbjct  7970  NNNNNNNNNNNNNNNNNNNNNNNNNNNNNNNNNNNNNNNNNNNNNNNNNNNNNNNNNNNN  8029

Query  8035  ACTGTTGTTAAAATTCGATCATTCATTATTCGCTGCATGAATTAGCTTGGCTGCAGGTCG  8094
                                                   ||||||||||||||||||||||
Sbjct  8030  NNNNNNNNNNNNNNNNNNNNNNNNNNNNNNNNNNN---GAATTAGCTTGGCTGCAGGTCG  8086

Query  8095  ACCTCGAGGGGCCGCCACCGCGGTGGAGCTCCAATTCGCCCTATAGTGAGTCGTATTACA  8154
             ||||||||||||||||||||||||||||||||||||||||||||||||||||||||||| 
Sbjct  8087  ACCTCGAGGGGCCGCCACCGCGGTGGAGCTCCAATTCGCCCTATAGTGAGTCGTATTACG  8146

Query  8155  AT---TCACTGGCCGTCGTTTTACAACGTCGTGACTGGGAAAACCCTGGCGTTACCCAAC  8211
                  |||||||||||||||||||||||||||||||||||||||||||||||||||||||
Sbjct  8147  CGCGCTCACTGGCCGTCGTTTTACAACGTCGTGACTGGGAAAACCCTGGCGTTACCCAAC  8206

Query  8212  TTAATCGCCTTGCAGCACATCCCCCTTTCGCCAGCTGGCGTAATAGCGAAGAGGCCCGCA  8271
             ||||||||||||||||||||||||||||||||||||||||||||||||||||||||||||
Sbjct  8207  TTAATCGCCTTGCAGCACATCCCCCTTTCGCCAGCTGGCGTAATAGCGAAGAGGCCCGCA  8266
```

```
Query  8272 CCGATCGCCCTTCCCAACAGTTGCGCAGCCTGAATGGCGAATGGAAATTGTAAGCGTTAA  8331
            ||||||||||||||||||||||||||||||||||||||||||||||||||||||||||||
Sbjct  8267 CCGATCGCCCTTCCCAACAGTTGCGCAGCCTGAATGGCGAATGGAAATTGTAAGCGTTAA  8326

Query  8332 TATTTTGTTAAAATTCGCGTTAAATTTTTGTTAAATCAGCTCATTTTTTAACCAATAGGC  8391
            ||||||||||||||||||||||||||||||||||||||||||||||||||||||||||||
Sbjct  8327 TATTTTGTTAAAATTCGCGTTAAATTTTTGTTAAATCAGCTCATTTTTTAACCAATAGGC  8386

Query  8392 CGAAATCGGCAAAATCCCTTATAAATCAAAAGAATAGACCGAGATAGGGTTGAGTGTTGT  8451
            ||||||||||||||||||||||||||||||||||||||||||||||||||||||||||||
Sbjct  8387 CGAAATCGGCAAAATCCCTTATAAATCAAAAGAATAGACCGAGATAGGGTTGAGTGTTGT  8446

Query  8452 TCCAGTTTGGAACAAGAGTCCACTATTAAAGAACGTGGACTCCAACGTCAAAGGGCGAAA  8511
            ||||||||||||||||||||||||||||||||||||||||||||||||||||||||||||
Sbjct  8447 TCCAGTTTGGAACAAGAGTCCACTATTAAAGAACGTGGACTCCAACGTCAAAGGGCGAAA  8506

Query  8512 AACCGTCTATCAGGGCGATGGCCCACTACGTGAACCATCACCCTAATCAAGTTTTTTGGG  8571
            ||||||||||||||||||||||||||||||||||||||||||||||||||||||||||||
Sbjct  8507 AACCGTCTATCAGGGCGATGGCCCACTACGTGAACCATCACCCTAATCAAGTTTTTTGGG  8566

Query  8572 GTCGAGGTGCCGTAAAGCACTAAATCGGAACCCTAAAGGGAGCCCCCGATTTAGAGCTTG  8631
            ||||||||||||||||||||||||||||||||||||||||||||||||||||||||||||
Sbjct  8567 GTCGAGGTGCCGTAAAGCACTAAATCGGAACCCTAAAGGGAGCCCCCGATTTAGAGCTTG  8626

Query  8632 ACGGGGAAAGCCGGCGAACGTGGCGAGAAAGGAAGGGAAGAAAGCGAAAGGAGCGGGCGC  8691
            ||||||||||||||||||||||||||||||||||||||||||||||||||||||||||||
Sbjct  8627 ACGGGGAAAGCCGGCGAACGTGGCGAGAAAGGAAGGGAAGAAAGCGAAAGGAGCGGGCGC  8686

Query  8692 TAGGGCGCTGGCAAGTGTAGCGGTCACGCTGCGCGTAACCACCACACCCGCCGCGCTTAA  8751
            ||||||||||||||||||||||||||||||||||||||||||||||||||||||||||||
Sbjct  8687 TAGGGCGCTGGCAAGTGTAGCGGTCACGCTGCGCGTAACCACCACACCCGCCGCGCTTAA  8746

Query  8752 TGCGCCGCTACAGGGCGCGTCAGGTGGCACTTTTCGGGGAAATGTGCGCGGAACCCCTAT  8811
            ||||||||||||||||||||||||||||||||||||||||||||||||||||||||||||
Sbjct  8747 TGCGCCGCTACAGGGCGCGTCAGGTGGCACTTTTCGGGGAAATGTGCGCGGAACCCCTAT  8806

Query  8812 TTGTTTATTTTTCTAAATACATTCAAATATGTATCCGCTCATGAGACAATAACCCTGATA  8871
            ||||||||||||||||||||||||||||||||||||||||||||||||||||||||||||
Sbjct  8807 TTGTTTATTTTTCTAAATACATTCAAATATGTATCCGCTCATGAGACAATAACCCTGATA  8866

Query  8872 AATGCTTCAATAATATTGAAAAAGGAAGAGTATGAGTATTCAACATTTCCGTGTCGCCCT  8931
            ||||||||||||||||||||||||||||||||||||||||||||||||||||||||||||
Sbjct  8867 AATGCTTCAATAATATTGAAAAAGGAAGAGTATGAGTATTCAACATTTCCGTGTCGCCCT  8926

Query  8932 TATTCCCTTTTTTGCGGCATTTTGCCTTCCTGTTTTTGCTCACCCAGAAACGCTGGTGAA  8991
            ||||||||||||||||||||||||||||||||||||||||||||||||||||||||||||
Sbjct  8927 TATTCCCTTTTTTGCGGCATTTTGCCTTCCTGTTTTTGCTCACCCAGAAACGCTGGTGAA  8986

Query  8992 AGTAAAAGATGCTGAAGATCAGTTGGGTGCACGAGTGGGTTACATCGAACTGGATCTCAA  9051
            ||||||||||||||||||||||||||||||||||||||||||||||||||||||||||||
Sbjct  8987 AGTAAAAGATGCTGAAGATCAGTTGGGTGCACGAGTGGGTTACATCGAACTGGATCTCAA  9046

Query  9052 CAGCGGTAAGATCCTTGAGAGTTTTCGCCCCGAAGAACGTTTTCCAATGATGAGCACTTT  9111
            ||||||||||||||||||||||||||||||||||||||||||||||||||||||||||||
Sbjct  9047 CAGCGGTAAGATCCTTGAGAGTTTTCGCCCCGAAGAACGTTTTCCAATGATGAGCACTTT  9106
```

```
Query  9112  TAAAGTTCTGCTATGTGGCGCGGTATTATCCCGTATTGACGCCGGGCAAGAGCAACTCGG  9171
             ||||||||||||||||||||||||||||||||||||||||||||||||||||||||||||
Sbjct  9107  TAAAGTTCTGCTATGTGGCGCGGTATTATCCCGTATTGACGCCGGGCAAGAGCAACTCGG  9166

Query  9172  TCGCCGCATACACTATTCTCAGAATGACTTGGTTGAGTACTCACCAGTCACAGAAAAGCA  9231
             ||||||||||||||||||||||||||||||||||||||||||||||||||||||||||||
Sbjct  9167  TCGCCGCATACACTATTCTCAGAATGACTTGGTTGAGTACTCACCAGTCACAGAAAAGCA  9226

Query  9232  TCTTACGGATGGCATGACAGTAAGAGAATTATGCAGTGCTGCCATAACCATGAGTGATAA  9291
             ||||||||||||||||||||||||||||||||||||||||||||||||||||||||||||
Sbjct  9227  TCTTACGGATGGCATGACAGTAAGAGAATTATGCAGTGCTGCCATAACCATGAGTGATAA  9286

Query  9292  CACTGCGGCCAACTTACTTCTGACAACGATCGGAGGACCGAAGGAGCTAACCGCTTTTTT  9351
             ||||||||||||||||||||||||||||||||||||||||||||||||||||||||||||
Sbjct  9287  CACTGCGGCCAACTTACTTCTGACAACGATCGGAGGACCGAAGGAGCTAACCGCTTTTTT  9346

Query  9352  GCACAACATGGGGGATCATGTAACTCGCCTTGATCGTTGGGAACCGGAGCTGAATGAAGC  9411
             ||||||||||||||||||||||||||||||||||||||||||||||||||||||||||||
Sbjct  9347  GCACAACATGGGGGATCATGTAACTCGCCTTGATCGTTGGGAACCGGAGCTGAATGAAGC  9406

Query  9412  CATACCAAACGACGAGCGTGACACCACGATGCCTGTAGCAATGGCAACAACGTTGCGCAA  9471
             ||||||||||||||||||||||||||||||||||||||||||||||||||||||||||||
Sbjct  9407  CATACCAAACGACGAGCGTGACACCACGATGCCTGTAGCAATGGCAACAACGTTGCGCAA  9466

Query  9472  ACTATTAACTGGCGAACTACTTACTCTAGCTTCCCGGCAACAATTAATAGACTGGATGGA  9531
             ||||||||||||||||||||||||||||||||||||||||||||||||||||||||||||
Sbjct  9467  ACTATTAACTGGCGAACTACTTACTCTAGCTTCCCGGCAACAATTAATAGACTGGATGGA  9526

Query  9532  GGCGGATAAAGTTGCAGGACCACTTCTGCGCTCGGCCCTTCCGGCTGGCTGGTTTATTGC  9591
             ||||||||||||||||||||||||||||||||||||||||||||||||||||||||||||
Sbjct  9527  GGCGGATAAAGTTGCAGGACCACTTCTGCGCTCGGCCCTTCCGGCTGGCTGGTTTATTGC  9586

Query  9592  TGATAAATCTGGAGCCGGTGAGCGTGGGTCTCGCGGTATCATTGCAGCACTGGGGCCAGA  9651
             ||||||||||||||||||||||||||||||||||||||||||||||||||||||||||||
Sbjct  9587  TGATAAATCTGGAGCCGGTGAGCGTGGGTCTCGCGGTATCATTGCAGCACTGGGGCCAGA  9646

Query  9652  TGGTAAGCCCTCCCGTATCGTAGTTATCTACACGACGGGGAGTCAGGCAACTATGGATGA  9711
             ||||||||||||||||||||||||||||||||||||||||||||||||||||||||||||
Sbjct  9647  TGGTAAGCCCTCCCGTATCGTAGTTATCTACACGACGGGGAGTCAGGCAACTATGGATGA  9706

Query  9712  ACGAAATAGACAGATCGCTGAGATAGGTGCCTCACTGATTAAGCATTGGTAACTGTCAGA  9771
             ||||||||||||||||||||||||||||||||||||||||||||||||||||||||||||
Sbjct  9707  ACGAAATAGACAGATCGCTGAGATAGGTGCCTCACTGATTAAGCATTGGTAACTGTCAGA  9766

Query  9772  CCAAGTTTACTCATATATACTTTAGATTGATTTAAAACTTCATTTTTAATTTAAAAGGAT  9831
             ||||||||||||||||||||||||||||||||||||||||||||||||||||||||||||
Sbjct  9767  CCAAGTTTACTCATATATACTTTAGATTGATTTAAAACTTCATTTTTAATTTAAAAGGAT  9826

Query  9832  CTAGGTGAAGATCCTTTTTGATAATCTCATGACCAAAATCCCTTAACGTGAGTTTTCGTT  9891
             ||||||||||||||||||||||||||||||||||||||||||||||||||||||||||||
Sbjct  9827  CTAGGTGAAGATCCTTTTTGATAATCTCATGACCAAAATCCCTTAACGTGAGTTTTCGTT  9886

Query  9892  CCACTGAGCGTCAGACCCCGTAGAAAAGATCAAAGGATCTTCTTGAGATCCTTTTTTTCT  9951
             ||||||||||||||||||||||||||||||||||||||||||||||||||||||||||||
Sbjct  9887  CCACTGAGCGTCAGACCCCGTAGAAAAGATCAAAGGATCTTCTTGAGATCCTTTTTTTCT  9946
```

```
Query  9952 GCGCGTAATCTGCTGCTTGCAAACAAAAAAACCACCGCTACCAGCGGTGGTTTGTTTGCC  10011
            ||||||||||||||||||||||||||||||||||||||||||||||||||||||||||||
Sbjct  9947 GCGCGTAATCTGCTGCTTGCAAACAAAAAAACCACCGCTACCAGCGGTGGTTTGTTTGCC  10006

Query  10012 GGATCAAGAGCTACCAACTCTTTTTCCGAAGGTAACTGGCTTCAGCAGAGCGCAGATACC  10071
             ||||||||||||||||||||||||||||||||||||||||||||||||||||||||||||
Sbjct  10007 GGATCAAGAGCTACCAACTCTTTTTCCGAAGGTAACTGGCTTCAGCAGAGCGCAGATACC  10066

Query  10072 AAATACTGTTCTTCTAGTGTAGCCGTAGTTAGGCCACCACTTCAAGAACTCTGTAGCACC  10131
             ||||||||| ||||||||||||||||||||||||||||||||||||||||||||||||||
Sbjct  10067 AAATACTGTCCTTCTAGTGTAGCCGTAGTTAGGCCACCACTTCAAGAACTCTGTAGCACC  10126

Query  10132 GCCTACATACCTCGCTCTGCTAATCCTGTTACCAGTGGCTGCTGCCAGTGGCGATAAGTC  10191
             ||||||||||||||||||||||||||||||||||||||||||||||||||||||||||||
Sbjct  10127 GCCTACATACCTCGCTCTGCTAATCCTGTTACCAGTGGCTGCTGCCAGTGGCGATAAGTC  10186

Query  10192 GTGTCTTACCGGGTTGGACTCAAGACGATAGTTACCGGATAAGGCGCAGCGGTCGGGCTG  10251
             ||||||||||||||||||||||||||||||||||||||||||||||||||||||||||||
Sbjct  10187 GTGTCTTACCGGGTTGGACTCAAGACGATAGTTACCGGATAAGGCGCAGCGGTCGGGCTG  10246

Query  10252 AACGGGGGGTTCGTGCACACAGCCCAGCTTGGAGCGAACGACCTACACCGAACTGAGATA  10311
             ||||||||||||||||||||||||||||||||||||||||||||||||||||||||||||
Sbjct  10247 AACGGGGGGTTCGTGCACACAGCCCAGCTTGGAGCGAACGACCTACACCGAACTGAGATA  10306

Query  10312 CCTACAGCGTGAGCTATGAGAAAGCGCCACGCTTCCCGAAGGGAGAAAGGCGGACAGGTA  10371
             ||||||||||||||||||||||||||||||||||||||||||||||||||||||||||||
Sbjct  10307 CCTACAGCGTGAGCTATGAGAAAGCGCCACGCTTCCCGAAGGGAGAAAGGCGGACAGGTA  10366

Query  10372 TCCGGTAAGCGGCAGGGTCGGAACAGGAGAGCGCACGAGGGAGCTTCCAGGGGGAAACGC  10431
             ||||||||||||||||||||||||||||||||||||||||||||||||||||||||||||
Sbjct  10367 TCCGGTAAGCGGCAGGGTCGGAACAGGAGAGCGCACGAGGGAGCTTCCAGGGGGAAACGC  10426

Query  10432 CTGGTATCTTTATAGTCCTGTCGGGTTTCGCCACCTCTGACTTGAGCGTCGATTTTTGTG  10491
             ||||||||||||||||||||||||||||||||||||||||||||||||||||||||||||
Sbjct  10427 CTGGTATCTTTATAGTCCTGTCGGGTTTCGCCACCTCTGACTTGAGCGTCGATTTTTGTG  10486

Query  10492 ATGCTCGTCAGGGGGGCGGAGCCTATGGAAAAACGCCAGCAACGCGGCCTTTTTACGGTT  10551
             ||||||||||||||||||||||||||||||||||||||||||||||||||||||||||||
Sbjct  10487 ATGCTCGTCAGGGGGGCGGAGCCTATGGAAAAACGCCAGCAACGCGGCCTTTTTACGGTT  10546

Query  10552 CCTGGCCTTTTGCTGGCCTTTTGCTCACATGTTCTTTCCTGCGTTATCCCCTGATTCTGT  10611
             ||||||||||||||||||||||||||||||||||||||||||||||||||||||||||||
Sbjct  10547 CCTGGCCTTTTGCTGGCCTTTTGCTCACATGTTCTTTCCTGCGTTATCCCCTGATTCTGT  10606

Query  10612 GGATAACCGTATTACCGCCTTTGAGTGAGCTGATACCGCTCGCCGCAGCCGAACGACCGA  10671
             ||||||||||||||||||||||||||||||||||||||||||||||||||||||||||||
Sbjct  10607 GGATAACCGTATTACCGCCTTTGAGTGAGCTGATACCGCTCGCCGCAGCCGAACGACCGA  10666

Query  10672 GCGCAGCGAGTCAGTGAGCGAGGAAGCGGAAGAGCGCCCAATACGCAAACCGCCTCTCCC  10731
             ||||||||||||||||||||||||||||||||||||||||||||||||||||||||||||
Sbjct  10667 GCGCAGCGAGTCAGTGAGCGAGGAAGCGGAAGAGCGCCCAATACGCAAACCGCCTCTCCC  10726

Query  10732 CGCGCGTTGGCCGATTCATTAATGCAGCTGGCACGACAGGTTTCCCGACTGGAAAGCGGG  10791
             ||||||||||||||||||||||||||||||||||||||||||||||||||||||||||||
Sbjct  10727 CGCGCGTTGGCCGATTCATTAATGCAGCTGGCACGACAGGTTTCCCGACTGGAAAGCGGG  10786
```

```
Query  10792 CAGTGAGCGCAACGCAATTAATGTGAGTTAGCTCACTCATTAGGCACCCCAGGCTTTACA  10851
             ||||||||||||||||||||||||||||||||||||||||||||||||||||||||||||
Sbjct  10787 CAGTGAGCGCAACGCAATTAATGTGAGTTAGCTCACTCATTAGGCACCCCAGGCTTTACA  10846

Query  10852 CTTTATGCTTCCGGCTCGTATGTTGTGTGGAATTGTGAGCGGATAACAATTTCACACAGG  10911
             ||||||||||||||||||||||||||||||||||||||||||||||||||||||||||||
Sbjct  10847 CTTTATGCTTCCGGCTCGTATGTTGTGTGGAATTGTGAGCGGATAACAATTTCACACAGG  10906

Query  10912 AAACAGCTATGACCATGATTACGCCAAGCTCGAAATTAACCCTCACTAAAGGGAACAAAA  10971
             ||||||||||||||||||||||||||||| || |||||||||||||||||||||||||||
Sbjct  10907 AAACAGCTATGACCATGATTACGCCAAGCGCGCAATTAACCCTCACTAAAGGGAACAAAA  10966

Query  10972 GCTGG-TACCCGCCCGGGGATCAGATCCGCGGCCGGCCGC--ATAGGCCACTAGTGGATC  11028
             ||||| |||||||||||| |||||||||||||||||||||  ||||||||||||||||||
Sbjct  10967 GCTGGGTACCCGCCCGGG-ATCAGATCCGCGGCCGGCCGCGCATAGGCCACTAGTGGATC  11025

Query  11029 TGGATCCTCTAGCTAGAGCTTTGCGTACTCGCAAATTATTAAAAATAAAACTTTAAAAAT  11088
             ||||||||||||||||||||||||||||||||||||||||||||||||||||||||||||
Sbjct  11026 TGGATCCTCTAGCTAGAGCTTTGCGTACTCGCAAATTATTAAAAATAAAACTTTAAAAAT  11085

Query  11089 AATTTCGTCTAATTAATATTATGAGTTAATTCAAACCCCACGGACATGCTAAGGGTTAAT  11148
             ||||||||||||||||||||||||||||||||||||||||||||||||||||||||||||
Sbjct  11086 AATTTCGTCTAATTAATATTATGAGTTAATTCAAACCCCACGGACATGCTAAGGGTTAAT  11145

Query  11149 CAACAATCATATCGCTGTCTCACTCAGACTCAATACGACACTCAGAATACTATTCCTTTC  11208
             ||||||||||||||||||||||||||||||||||||||||||||||||||||||||||||
Sbjct  11146 CAACAATCATATCGCTGTCTCACTCAGACTCAATACGACACTCAGAATACTATTCCTTTC  11205

Query  11209 ACTCGCACTTATTGCAAGCATACGTTAAGTGGATGTCTCTTGCCGACGGGACCACCTTAT  11268
             ||||||||||||||||||||||||||||||||||||||||||||||||||||||||||||
Sbjct  11206 ACTCGCACTTATTGCAAGCATACGTTAAGTGGATGTCTCTTGCCGACGGGACCACCTTAT  11265

Query  11269 GTTATTTCATCATG  11282
             ||||||||||||||
Sbjct  11266 GTTATTTCATCATG  11279
```
