# Supplementary material for: Expression Pattern of the AB1-Gal4 Driver in Drosophila Third-Instar Larvae
Source: Int J Mol Sci. 2025 Apr 22;26(9):3923. doi: 10.3390/ijms26093923 (PMC12071433; doi:10.3390/ijms26093923)
Supplement: Supplementary file 1 [file ijms-26-03923-s001.zip › ijms-3584700-supplementary.pdf]

Supplementary Materials

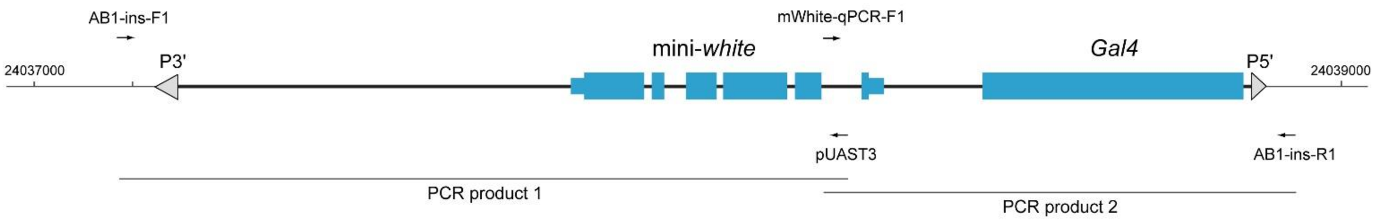

Figure S1. Insertion of the P{GawB} transposon in chromosome 3R in the AB1-Gal4 driver line. Thin and thick horizontal black lines show segments of chromosome 3R flanking the transposon insertion site and the transposon internal sequence, respectively. Genes on the reverse strand are shown in blue. Grey triangles represent the 5′ and 3′ *P*-element ends. The PCR products 1 and 2 are 7400 bp and 4784 bp in size, respectively.

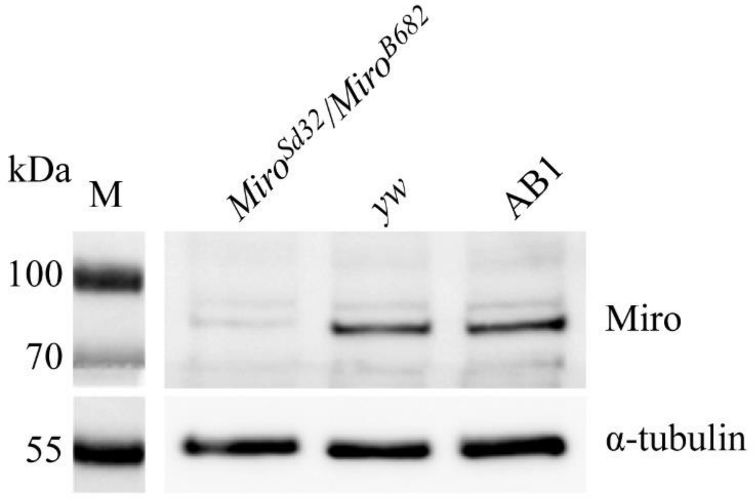

Figure S2. The level of the Miro protein in the third instar larval CNS from *Miro* mutants and AB1-Gal4 homozygotes. A representative Western blot of larval CNS with adjacent imaginal discs showing that, compared to *yw* control, the level of the Miro protein is significantly reduced in *Miro*^{Sd32}/*Miro*^{B682} mutants, but not in the AB1-Gal4 homozygotes (AB1). M—Prestained Protein Ladder. α-tubulin is a loading control.
